# RWRMTN: a tool for predicting disease-associated microRNAs based on a microRNA-target gene network

**DOI:** 10.1186/s12859-020-03578-3

**Published:** 2020-06-15

**Authors:** Duc-Hau Le, Trang T. H. Tran

**Affiliations:** Department of Computational Biomedicine, Vingroup Big Data Institute, No 7, Bang Lang 1 Street, Viet Hung Ward, Long Bien District, Hanoi, Vietnam

**Keywords:** Disease-associated miRNAs, miRNA-target interaction, Random walk with restart, Automation, Cytoscape app, CyREST command APIs, CyREST APIs

## Abstract

**Background:**

The misregulation of microRNA (miRNA) has been shown to cause diseases. Recently, we have proposed a computational method based on a random walk framework on a miRNA-target gene network to predict disease-associated miRNAs. The prediction performance of our method is better than that of some existing state-of-the-art network- and machine learning-based methods since it exploits the mutual regulation between miRNAs and their target genes in the miRNA-target gene interaction networks.

**Results:**

To facilitate the use of this method, we have developed a Cytoscape app, named RWRMTN, to predict disease-associated miRNAs. RWRMTN can work on any miRNA-target gene network. Highly ranked miRNAs are supported with evidence from the literature. They then can also be visualized based on the rankings and in relationships with the query disease and their target genes. In addition, automation functions are also integrated, which allow RWRMTN to be used in workflows from external environments. We demonstrate the ability of RWRMTN in predicting breast and lung cancer-associated miRNAs via workflows in Cytoscape and other environments.

**Conclusions:**

Considering a few computational methods have been developed as software tools for convenient uses, RWRMTN is among the first GUI-based tools for the prediction of disease-associated miRNAs which can be used in workflows in different environments.

## Background

Prediction of novel disease-associated miRNAs is an important task in biomedicine. A number of computational methods, including network-based and machine learning-based ones, have been introduced for predicting disease-associated miRNAs [1–3]. However, few of them have been developed as prediction tools to facilitate their use [4, 5]. In general, disease similarity, miRNA similarity and known disease-miRNA associations are usually used as input data for both approaches [6]. The similarity information was represented as networks (e.g., disease similarity network, miRNA similarity network) in network-based methods, then label propagation algorithms were often used to transfer disease-miRNA labels between miRNAs and diseases via the networks [7–13]. Meanwhile, that information was often represented by matrices in some machine learning-based methods, then matrix factorization methods were used [14–17]. Network-based methods are mainly relied on homogeneous miRNA networks [10–13]. In such networks, nodes represent miRNAs and edges represent the degree of functional similarity between miRNAs. Based on these homogeneous miRNA networks, associations between miRNAs and diseases are predicted based on the assumption that functionally related miRNAs associate with phenotypically similar diseases.

A common limitation of the homogeneous miRNA network-based methods is that the biological interactions between miRNAs and their target genes might be used ineffectively. This is because those interactions were only embedded as a degree of similarity among miRNAs. For instances, the similarity between two miRNAs was measured by a number of their shared target genes [11, 18–21]. These miRNA-target interactions, including both predicted and experimentally validated ones, are now largely available in a number of miRNA-target databases [22] and become useful resources for analysis. This has inspired us to propose a novel network-based method for predicting disease-associated miRNA [19] using miRNA-target gene interactions. More specifically, we exploited the mutual regulation between miRNAs and their target genes to construct mutual heterogeneous miRNA-target gene interaction networks (shortly called miRNA-target interaction networks) (i.e., nodes represent miRNAs and their target genes, and edges refer to the physical interactions between miRNAs and their target genes). We then proposed a novel method, namely RWRMTN, based on random walk with restart (RWR) algorithm on the miRNA-target interaction network to rank candidate miRNAs. Experiment results show that RWRMTN outperforms existing state-of-the-art methods including a network-based method RWRMDA [10], which also uses the RWR algorithm but on homogeneous miRNA networks, and a machine learning-based method RLSMDA [17]. RWRMTN was also proven to be stable, and it achieved relatively high performance for both experimentally validated and predicted miRNA-target gene networks [19].

Although many computational methods have been proposed for predicting disease-associated miRNAs, however, only a few of them have been developed as software tools which are convenient for biomedical applications. Indeed, we investigated 59 software tools for predicting disease-miRNA associations in OMICtools [5], but most of them provided only source code and with command-line interfaces, which are not convenient for most of the biologists. Ten of them provide web-based interfaces, but only a few are accessible such as CHNmiRD [23], OncomiR [24] and miRConnect [25] (Table 3 shows a comparison between RWRMTN with the ten web-based tools).

To facilitate the use of RWRMTN, we develop a tool running on the Cytoscape framework [33] to predict disease-associated miRNAs. RWRMTN can rank all miRNA candidates in the miRNA-target gene network as well as candidates provided by users. In addition, it can provide evidence from literature about associations with the disease of interest for highly ranked miRNAs. These miRNAs then can also be visualized in relationships with their target genes as well as supporting evidence. Equipped by newly-introduced automation features of Cytoscape, the use of RWRMTN can be extended to workflows in other environments. Functions of RWRMTN were demonstrated in predicting breast and lung cancer-associated miRNAs via workflows in Cytoscape GUI platform and other environments.

## Implementation

### Main functions and workflow

**Fig. 1:**
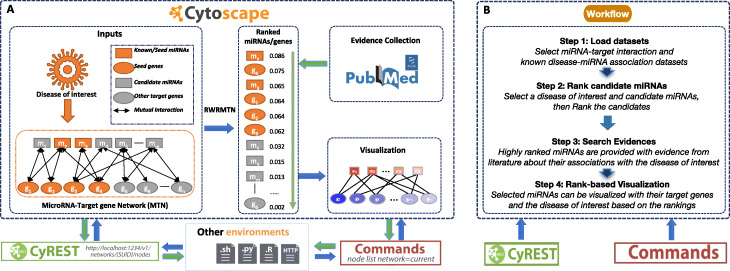
An illustration of RWRMTN app and workflow for predicting disease-associated miRNAs. **a** Given a disease of interest, RWRMTN app is run on a miRNA-target network to rank candidate miRNAs, then highly ranked miRNAs are supported with evidence from literature and visualized by their rankings. These functions of RWRMTN app can be called in workflows from other environments (e.g., Bash script, Python, R, HTTP) using automation features (e.g., CyREST API). **b** Prediction of novel miRNAs associated with a disease of interest (e.g., breast cancer) can be done through four steps

RWRMTN is written as a Cytoscape app (Fig. 1(a)). The main task of RWRMTN is to predict novel disease-associated miRNAs. The primary result is ranking of candidate miRNAs based on our previously proposed method RWRMTN [19] (see brief description in the next section). To make the result more reliable and intuitive, RWRMTN provides two more functions which are evidence collection and visualization. The evidence collection function gathers evidence from literature about the association of highly ranked miRNAs and the disease of interest. The visualization function builds a network of the selected miRNAs (e.g., highly ranked miRNAs) in relationships with their target genes and the disease of interest. To make RWRMTN reaches a wider range of users, we additionally implement automation features so that those functions of RWRMTN can be called using CyREST APIs and CyREST Command APIs from workflows written in other environments such as R and Python.

Prediction of novel disease-associated miRNAs is designed to complete through a four-step workflow (Fig. 1(b)). First, two datasets, including a miRNA-target interaction network and known disease-miRNA associations, must be specified. To facilitate the use of RWRMTN, we preinstalled some widely used datasets. However, to be flexible, the user can freely import others. Second, a disease of interest and candidate miRNAs are selected to rank. The candidates can be either all remaining miRNAs in the miRNA-target interaction network (i.e., excluding known miRNAs associated with the disease of interest) or freely inputted by the user. Third, highly ranked candidate miRNAs can be selected for an evidence search. More specifically, we searched for the co-occurrence of a candidate miRNA and the disease of interest from literature in PubMed via NCBI API [34]. Thus, relevant evidence from newly published research will be retrieved each time of the evidence search. Evidenced miRNAs are provided with PubMed ID of the studies supporting an association between the miRNAs and the disease of interest. Finally, the selected miRNAs can be visualized based on their rankings and in relation with their target genes, the disease of interest and the supporting PubMed IDs.

### RWRMTN method

RWR is a variant of random walk algorithm, and it mimics a walker that moves from a current node in a network (i.e.*,* G(*V*, *E*) with a set of nodes *V* = {*v*_*1*_*, v*_*2*_*, …, v*_*N*_} and a set of links *E* = {(*v*_*i*_*, v*_*j*_)| *v*_*i*_*, v*_*j*_∈*V*}, a set of seed nodes *S* ⊆ *V*) to a randomly selected adjacent node or goes back to source nodes (also called seed nodes) with a restart-probability (*γ*).

1 $$ {p}_{t+1}=\left(1-\gamma \right){W}^{\prime }{p}_t+\gamma {p}_0 $$

where:

- *W′* represents a transition probability matrix of the network
- *p*_t_ is a |*V*| × *1* probability vector of |*V*| nodes at a time step *t* of which the *i*th element represents the probability of the walker being at node *v*_*i*_∈*V*.
- *p*_*0*_ is the |*V*| × *1* initial probability vector.

In RWRMDA method [10], the RWR algorithm was used to rank miRNAs in homogeneous miRNA networks; therefore, the set of seed nodes (*S*) only contains known disease miRNA (*S*_*m*_) (i.e., *S* = *S*_*m*_).

2 $$ {\left({p}_0\right)}_i=\left\{\begin{array}{c}\frac{1}{\left|{S}_m\right|}\ {ifv}_i\in {S}_m\\ {}\ 0\  otherwise\end{array}\right. $$

In RWRMTN [19], we assumed that the mutual regulation between a miRNA and their targets leads to a transfer of disease information between them. Therefore, we force the RWR algorithm to start from a set of seed nodes which consists of not only known disease miRNAs but also their target genes. In particular, we enlarge the set of seed node (*S*) by adding target genes (*S*_*g*_) of the known disease miRNAs (i.e., *S*=*S*_*m*_∪*S*_*g*_), with *α*∈ (0, 1) is a weight parameter, which controls the disease information transferred between miRNAs and their target genes.

3 $$ {\left({p}_0\right)}_i=\left\{\begin{array}{c}\alpha \frac{1}{\left|{S}_m\right|}\ {ifv}_i\in {S}_m\\ {}\left(1-\alpha \right)\frac{1}{\left|{S}_g\right|}\ {ifv}_i\in {S}_g\\ {}0\  otherwise\end{array}\right. $$

For both methods, all miRNAs in the network are eventually ranked according to the steady-state probability vector *p*_*∞*_, which is obtained by repeating the iterations until convergence is reached (in this study, ||*p*_t + 1_-*p*_t_|| < 10^− 6^).

In the previous study, we have investigated the effects of the restart-probability (*γ*) and the weight parameter (*α*) on the prediction performance of RWRMTN on two miRNA-target gene networks. More specifically, we varied the weight parameter (α) in the range {0.1, 0.3, 0.5, 0.7, 0.9} and the restart probability *γ* in the range [0.1, 0.9] in steps of 0.1. Experimental results showed that the performance of RWRMTN slightly increased according to the change of the weight parameter in both the networks. This indicates that disease information contained in known disease miRNAs is still more important than that in their target genes when ranking candidate disease-associated miRNAs. In addition, RWRMTN is either slightly better or stable when the restart probability *γ* increased in the two networks. The slight increase in the prediction performance in a network suggested that disease miRNAs in that network are less modularized than the other.

### Preinstalled data

To facilitate the use of the app, we preinstalled some datasets. First, two miRNA-target gene networks collected from an experimentally validated dataset, miRWalk [35] and a predicted dataset, TargetScan [36] were preinstalled (Table 1). However, the user can import any other miRNA-target gene network to use. Second, two known disease-miRNA association datasets were also pre-collected, i.e., miR2Disease [37] and HMDD [38] (Table 2).

**Table 1:** Preinstalled miRNA-target gene interaction datasets

Datasets	Number of miRNAs	Number of target genes	Number of known interactions
miRWalk	740	11,976	38,569
TargetScan	1537	15,031	520,256

**Table 2:** Preinstalled known disease-miRNA association datasets

Datasets	Number of miRNAs	Number of diseases	Number of known associations
miR2Disease	118	53	270
HMDD	574	243	5618

## Results

**Fig. 2:**
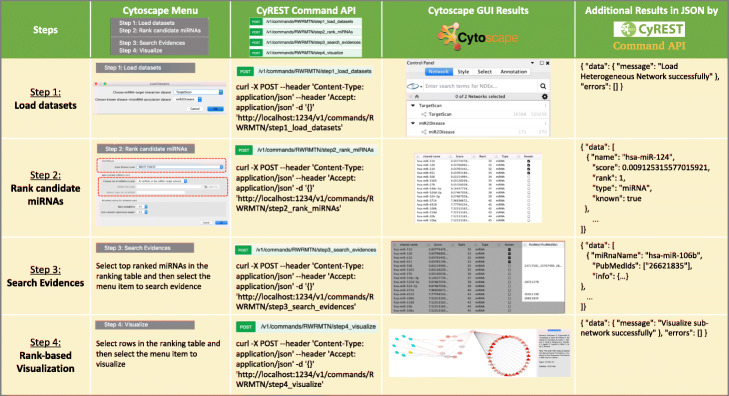
Prediction performance of breast cancer by RWRMTN on the two miRNA-target gene datasets (miRWalk and TargetScan). **a** The AUC values when the restart probability was varied in a range [0.1, 0.9]. **b** The ROC curve and the AUC values when the restart probability was set to 0.5 for the case study. For all experiments, the weight parameter was set to 0.5

**Fig. 3:**
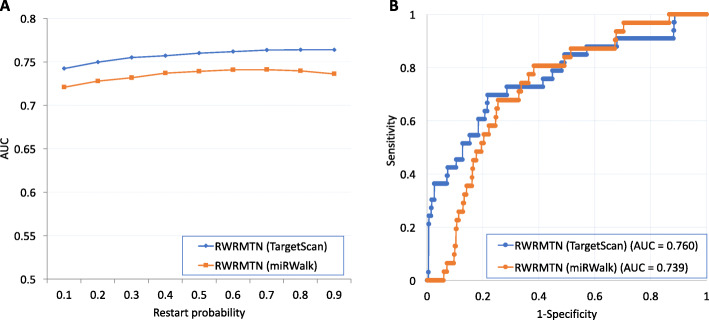
A four-step workflow of RWRMTN for predicting breast cancer-associated miRNAs. *Step1: Load datasets*: a miRNA-target interaction dataset and a known disease-miRNA association dataset must be selected. *Step 2: Rank candidate miRNAs*: a disease of interest (e.g., breast cancer) is chosen, then a set of candidate miRNAs is specified and ranked. *Step 3: Search Evidences*: Top-ranked candidate miRNAs are selected and provided with evidence from literature about their associations with the disease of interest. *Step 4: Rank-based visualization*: the selected candidate miRNAs are visualized based on their rankings and their relationships with the disease of interest, known breast cancer-associated miRNAs and supporting PubMed IDs. These steps can be performed by either the Cytoscape menu or CyREST Command APIs called from other environments (e.g., R)

The overall prediction performance of RWRMTN on a set of diseases was reported in our previous study [19]. In this section, the effectiveness of RWRMTN was demonstrated for breast cancer in two case studies via different scenarios. Given the breast cancer with their known associated miRNAs, firstly, all other miRNAs in the miRNA-target networks were used as candidates. The overall performance of RWRMTN in terms of AUC (Area under the ROC curve) for breast cancer was assessed using a leave-one-out cross-validation (LOOCV) scheme (Fig. 2). Then, the candidates were ranked and supported with evidence. More specifically, we performed this task with candidate miRNAs from the miRNA-target gene network constructed from TargetScan [36] based on 31 known breast cancer-associated miRNAs reported in miR2Disease [37] using Cytoscape menu and Command APIs via the four-step workflow (Fig. 3). As a result, four of top 10 miRNA candidates were supported with evidence from literature about their associations with breast cancer. Secondly, only miRNAs that were differentially expressed between case and control samples were used as the candidates for ranking. More specifically, we ranked 799 miRNAs that were differentially expressed between the 64 wild-type samples and 36 *TP53* mutant breast cancer samples collected from [39] via CyREST API called in R environment using the miRNA-target gene network constructed from miRWalk [35] and the known disease-miRNA association dataset HMDD [38]. The scripts for calling the CyREST API in other environments such as Python and Bash were also introduced. Furthermore, another case study on lung cancer was demonstrated (See more detail in Additional file 1).

### Comparison between RWRMTN with other tools

We investigated 59 software tools (as of 16th March 2020) for predicting disease-miRNA associations in OMICtools [5] which has accumulated tens of thousands of tools for omics data. However, most of them provide only source code and with command-line interfaces, which are not convenient for most of the biologists. Indeed, 14 out of the 59 tools are web-based including: mirfluence [26], Ifmda [27], CHNmiRD [23], miRiaD [28], OncomiR [24], miRPD [40], MIDP [30], DISMIRA [31], MDHGI [32], CMP [29], miRConnect [25], DMPred [41], MMiRNA-Tar [42] and miRNACon [43]. However, the web page of DMPred [41] could not be found, and miRPD [40] was actually designed for identifying microRNAs from deep sequencing data. In addition, MMiRNA-Tar [42] and miRNACon [43] were not designed specifically for predicting disease-associated miRNAs. Thus, the four tools were eliminated from the comparison. Thus, we finally have compared the rest (ten tools) with RWRMTN (Table 3).

**Table 3:** A comparison between RWRMTN and other tools for predicting disease-associated miRNAs

Tools	Function				Others		Website	Accessible
	*Ranking*	*Evidence search*	*Visualize*	*Automation*	*Multiple diseases*	*User-defined datasets*		
**RWRMTN**	✓	✓	✓	✓	✓	✓	*http://apps.cytoscape.org/apps/rwrmtn*	✓
**miRfluence** [26]	✓		✓		✓		*http://bnet.egr.vcu.edu/mirfluence*	
**Ifmda** [27]	✓				✓		*http://lab.malab.cn/soft/ifmda*	
**CHNmiRD** [23]	✓				✓		*http://www.bio-bigdata.com/CHNmiRD*	✓
**miRiaD** [28]	✓	✓			✓		*http://biotm.cis.udel.edu/miRiaD*	
**OncomiR** [24]	✓	✓			Cancer		*http://www.oncomir.org*	✓
CMP [29]	✓	✓			Cancer		*http://bioinfo.hrbmu.edu.cn/CMP*	
**MIDP** [30]	✓				✓		*http://bioinfolab.stx.hk/midp/*	
**DISMIRA** [31]	✓		✓		✓		*http://bnet.egr.vcu.edu:8080/dismira*	
**MDHGI** [32]	✓	✓			✓		*http://chengroup.cumt.edu.cn/tool/mdhgi/*	
**miRConnect** [25]	✓				Cancer		*http://mirconnect.org:8002/miRConnect/*	✓

In the aspect of function comparison to RWRMTN, as tools for predicting disease-associated miRNAs, all of them provided ranking for candidate miRNAs, however only four of them provided evidence for potential disease-miRNA associations. RWRMTN, on the other hand, integrated NCBI API [34] to the evidence search function, thus relevant evidence from recently published research will be retrieved each time users perform the task. Besides displaying the results in tabular form, only two tools visualized the association between miRNAs and diseases. In contrast, highly ranked miRNAs, known miRNAs, target genes, the disease of interest as well as supporting PubMed IDs and their associations could be intuitively viewed in a network in RWRMTN. Lastly, none of them provides automation function of reproducibility to different environments. Equipped with CyREST API of Cytoscape platform, RWRMTN functions can be invoked from any workflows running in other environments. In other aspects, like most of the tools, RWRMTN was also designed for multiple diseases. However, it is more flexible in providing the input data, since users can opt between preinstalled datasets or their own. None of the web-based tools provided this function. Finally, only three out of the ten web-based tools can be accessed via links provided in their publications. In contrast, RWRMTN is easy to be managed and maintained since the available update of RWRMTN will be notified by Cytoscape.

### Prediction of breast cancer-associated miRNAs using Cytoscape menu and CyREST command API

**Fig. 4:**
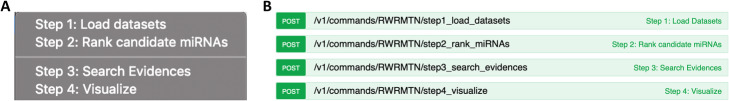
Perform the four-step workflow of RWRMTN. **a** via Cytoscape menu (Apps ➔ RWRMTN). **b** via CyREST Command APIs (Help ➔ Automation ➔ CyREST Command API ➔ RWRMTN)

In this section, we demonstrate the use of RWRMTN in predicting novel breast cancer-associated miRNAs by the four-step workflow. The workflow can be accomplished using either Cytoscape menu or CyREST Command API (Fig. 4) (See more detail in Additional file 1).

For the first step, a miRNA-target interaction dataset TargetScan [36] and a known disease-miRNA association dataset miR2Disease [37] were used. Note that, besides the two preinstalled datasets, miRNA-target interaction network can be freely imported by the user.

For the second step, a set of candidate miRNAs for breast cancer (OMIM ID: 114480) is ranked. To this end, we first selected the disease, then all miRNAs in the selected miRNA-target interaction network were selected as candidates for ranking. Note that, users can manually input candidate miRNAs or import them from a file. After that, the candidate miRNAs were ranked by the RWRMTN method [19] with default parameter settings (See more detail in Additional file 1).

**Fig. 5:**
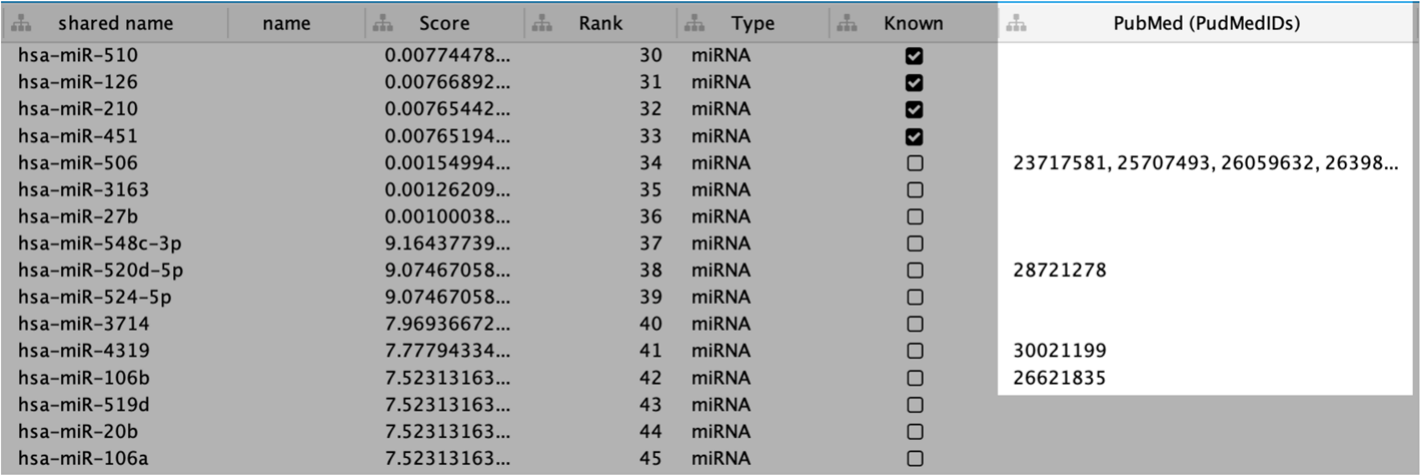
Ranked candidate miRNAs. Top-ranked candidate miRNAs were selected and provided with evidence from the literature (highlighted part)

For the third step, we selected the top ten ranked candidate miRNAs, then find the evidence of their associations with breast cancer from literature (PubMed). As a result, four of them were supported with evidence from literature about their associations with breast cancer (Fig. 5).

For example, *hsa-miR-506* was supported by five studies (PubMed IDs: 23717581, 25707493, 26059632, 26398880 and 27542202). The study (PubMed ID: 23717581) [44] showed that has-miR-506 regulates epithelial-mesenchymal transition in breast cancer cell lines. Meanwhile, the study (PubMed ID: 26059632) [45] proved notable inhibition of *hsa-miR-506* over-expression to proliferation and metastasis of breast cancer cells. In addition, study (PubMed ID: 26398880) [46] indicated that the mechanism underlying miRNA-506 is a contributing factor in breast carcinogenesis (*has-miR-506* was proven to be a tumor suppressor). In addition, *hsa-miR-520d-5p* supported by a study (PubMed ID: 28721278) [47]. More specifically, it was reported that this miRNA upregulates the activation of BRCA1 (breast cancer 1, early onset) in the DNA repair process – 35 days after transfection. Moreover, *hsa-miR-4319* was showed in study PubMed ID: 30021199 as a suppressor of the malignancy of triple-negative breast cancer by regulating self-renewal and tumorigenesis of stem cells. Finally, *hsa-miR-106b* was proven by the experiment carried on patient samples and cell lines in the study (PubMed ID: 26621835) [48].

**Fig. 6:**
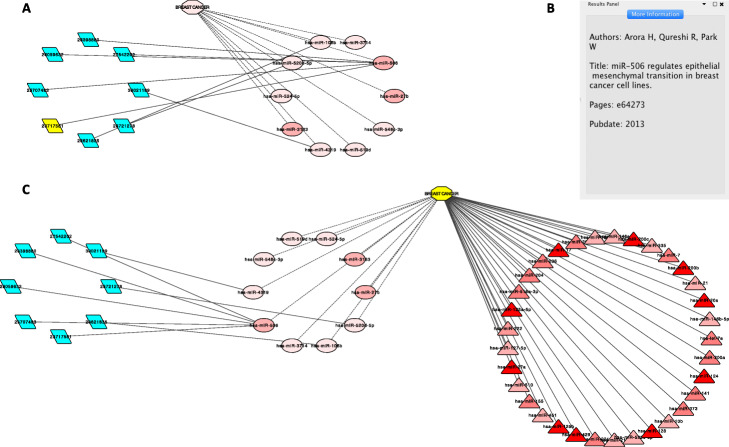
Visualization of top-ranked candidate miRNAs and breast cancer. **a** With supporting PubMed IDs and (**b**) detail information of the selected PubMed ID. **c** With known breast cancer-associated miRNAs. PubMed IDs, disease, candidate and known miRNAs are represented in parallelogram, octagon, ellipse and triangle shapes, respectively. The darker red a miRNA is the higher ranking it has. Known and candidate associations with the disease are represented by solid and dashed lines, respectively

For the final step, the top selected candidate miRNAs can be visualized in a network based on the rankings. In addition, target genes, the disease of interest and detail information of PubMed IDs collected from Step 3 such as *paper title, author list, journal name* can be displayed aside in this network (Fig. 6).

### Prediction of breast cancer-associated miRNAs by calling CyREST API

In this section, we first introduce some developed CyREST APIs, which provides some helpful functions. Second, we demonstrate their use in a workflow in R environment.

**Fig. 7:**
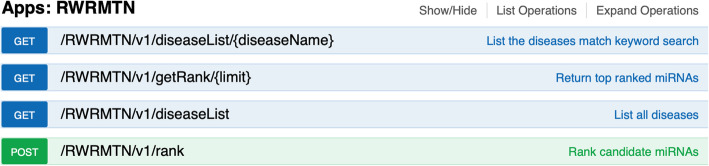
CyREST APIs of RWRMTN. The four APIs are used to query from datasets in the app (GET) and to let the app rank candidate miRNAs (POST)

A total of four CyREST APIs were developed in RWRMTN (Fig. 7): First, GET /RWRMTN/v1/diseaseList returns a list of all diseases (OMIM ID and disease name) available in the selected known disease-miRNA association database (e.g., HMDD [38]). Based on this list, users can select a disease of interest. Second, GET /RWRMTN/v1/diseaseList/{diseaseName} provides a list of diseases whose names match the query {diseaseName} parameter (e.g., breast). This API helps user narrow down the list of diseases to the disease of interest (e.g., OMIM ID 114480 for breast cancer). Third, POST /RWRMTN/v1/rank lets RWRMTN rank candidate miRNAs. Finally, GET:/RWRMTN/v1/getRank/{limit} returns top-ranked miRNAs by setting {limit} parameter.

In this case study, we used a dataset GSE19783 from a study [39] published in NCBI GEO [49], which was created using Agilent-019118 Human miRNA Microarray 2.0 G4470B platform (GPL8227) and Agilent-014850 Whole Human Genome Microarray 4x44K G4112F (GPL6480) (https://www.ncbi.nlm.nih.gov/geo/query/acc.cgi?acc=GSE19783). The study characterizes breast cancer subtypes from joint analysis of high throughput miRNA (using GPL8227) and mRNA (using GPL6480) data.

In this case study, we identified 799 candidate miRNAs which were differentially expressed between the 64 wild-type samples (WT) and 36 *TP53* mutant samples. Then, we ranked the candidate miRNAs by RWRMTN via a CyREST API using a miRNA-target interaction dataset miRWalk [35] and a known disease-miRNA association dataset HMDD [38] via workflow in R environment using CyREST API POST /RWRMTN/v1/rank (See more detail in Additional file 1).

## Conclusions

In this study, we introduce a tool as a Cytoscape app, RWRMTN, for predicting novel disease-associated miRNAs. The tool was developed based on our previously proposed method with the same name, which was proven to be better than other state-of-the-art methods on overall prediction performance and to have the ability in predicting novel miRNAs associated with 23 diseases [19]. Because the core method is a network-based, thus RWRMTN can exploit network integration and visualization functions of Cytoscape. In particular, the tool relies on miRNA-target gene networks to rank candidate miRNAs with supporting functions such as evidence collection and visualization for highly ranked candidate miRNAs. In addition, by implementing automation functions, it can be used in workflows in other environments. We further demonstrate the use of RWRMTN by showing its ability in predicting novel breast and lung cancer-associated miRNAs.

### Availability and requirements

**Project name:** RWRMTN

**Project home page:** https://github.com/hauldhut/RWRMTN

**Operating system(s):** Mac OS, Linux, Windows

**Programming language:** Java

**Other requirements:** JDK 1.8 or higher, Cytoscape 3.x or higher

**License:** Apache License

**Any restrictions to use by non-academics:** licence needed

## Supplementary information

**Additional file 1.** User manual & Case studies.

## Data Availability

RWRMTN is distributed as a Cytoscape app, and can be downloaded freely at Cytoscape App https://apps.cytoscape.org/apps/rwrmtn for non-commercial use.

## Abbreviations

GUI: Graphical User Interface
RWR: Random Walk with Restart
