# Supplementary material for: RWRMTN: a tool for predicting disease-associated microRNAs based on a microRNA-target gene network
Source: BMC Bioinformatics. 2020 Jun 15;21:244. doi: 10.1186/s12859-020-03578-3 (PMC7296691; doi:10.1186/s12859-020-03578-3)
Supplement: Supplementary file 1 — Additional file 1. User manual & Case studies. [file 12859_2020_3578_MOESM1_ESM.pdf]

RWRMTN: a tool for predicting disease-associated microRNAs based on a microRNA-target gene network

Duc-Hau Le[1,*], Trang T.H Tran[1]

[1]Department of Computational Biomedicine, Vingroup Big Data Institute, No 7, Bang Lang 1 Street, Viet Hung Ward, Long Bien District, Hanoi, Vietnam.

[*]To whom correspondence should be addressed (hauldhut@gmail.com).

Additional file 1

User Manual & Case studies

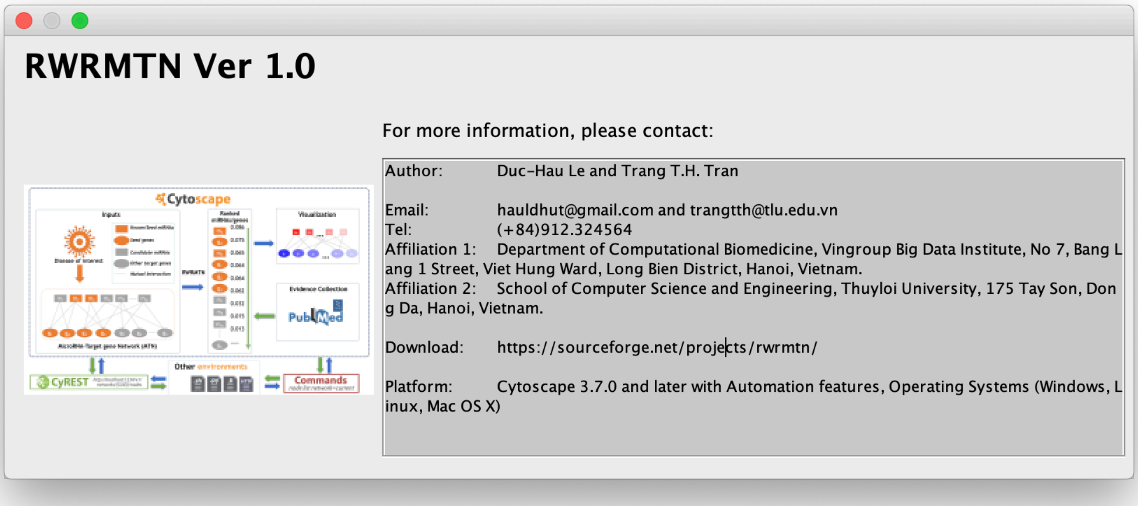

Table of Contents

I. Setup 3

1. Install Cytoscape 3
2. Install RWRMTN app 3

II. Overview of RWRMTN 4

III. Case study: Prediction of breast cancer-associated miRNAs 5

1. Run RWRMTN using Cytoscape menu and CyREST Command API 6
   - Step 1: Load datasets 6
   - Step 2: Rank candidate miRNAs 8
   - Step 3: Search Evidences 12
   - Step 4: Visualize 14
2. Run RWRMTN by calling CyREST API 16
   - Overview of CyREST APIs 16
   - Using RWRMTN in a workflow in R environment 19
   - Using RWRMTN in a workflow in other environments 22

IV. Case study: Prediction of lung cancer-associated miRNAs 24

- Step 1: Load datasets 24
- Step 2: Rank candidate miRNAs 24
- Step 3: Search Evidences 24
- Step 4: Visualize 25

V. Reference 26

I. Setup

1. Install Cytoscape

- RWRMTN1.0 can only run on **Cytoscape 3.6 (or later)** platform, **which has Automation features**, therefore user should download this version at http://cytoscape.org/
- Cytoscape need JRE to run, therefore download JRE version 8.x or later from http://www.oracle.com/technetwork/java/index.html and install it.
- Install Cytoscape to the root folder (e.g., /Applications/Cytoscape_v3.6.0).

2. Install RWRMTN app

There are two ways to install RWRMTN app.

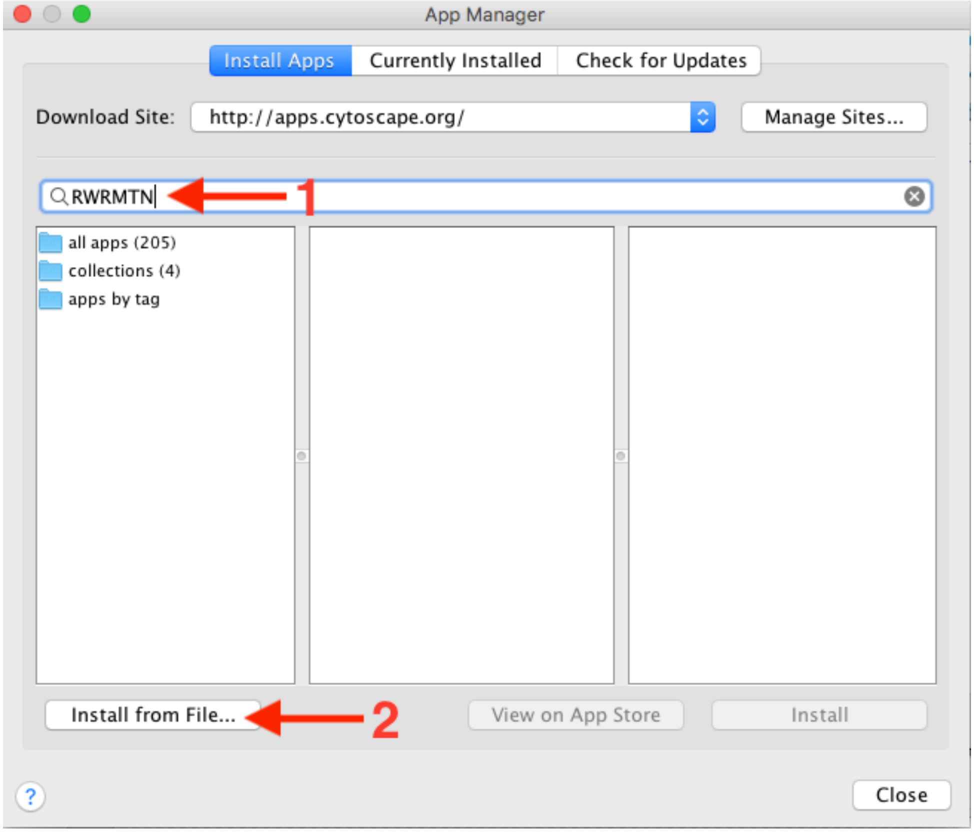

- *Method 1: Automatically install from Cytoscape Appstore:* Select menu **App → AppManager** in Cytoscape. Then type RWRMTN in search box to install directly from Appstore of Cytoscape.
- *Method 2: Manual install*:
  - Download RWRMTN_v1.0.jar file from https://github.com/hauldhut/RWRMTN
  - Then, install it by going to **Apps→ App Manager**…. After that, choose **Install from file…**, then browse the downloaded RWRMTN_v1.0.jar file.

Note that: RWRMTN_v1.0 can work on Windows, Ubuntu and Mac OS. The following manual was prepared when running RWRMTN on Mac OS.

II. Overview of RWRMTN

After installing, RWRMTN will be automatically loaded in the App menu of Cytoscape

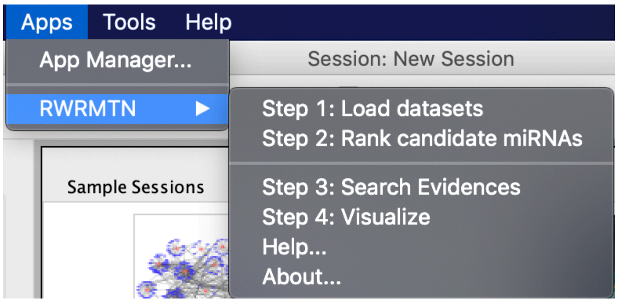

The main tasks (Rank candidate miRNAs, Evidence Search and Visualization) of RWRMTN are completed after four steps:

- **Step 1**: Load data sets (miRNA-target gene interactions and known disease-miRNA associations)
- **Step 2**: Rank candidate miRNAs (including 4 sub-steps)
    - 1. Select a disease of interest
    - 2. Input candidate miRNAs to rank
    - 3. Parameters setting (for advanced users)
    - 4. Rank
- **Step 3**: Search Evidences
- **Step 4**: Visualize

These steps can be performed, and results of each step can be exposed

- Using Cytoscape menu

Beside the Cytoscape GUI, new upgraded automation feature of Cytoscape allows functions of Cytoscape and apps called via REST API. Therefore, we can call the functions of Cytoscape and apps in workflows in other environments such as R, Python, etc... Thus, RWRMTN functions can be used by

- CyREST Command API
- CyREST API

III. Case study: Prediction of breast cancer-associated miRNAs

In this section, we demonstrate the use of RWRMTN in predicting novel breast cancer-associated miRNAs by following workflow. The workflow can be done using Cytoscape menu or CyREST Command API. In addition, intermediate results can be exposed from other environments (e.g., R statistics) using CyREST API.

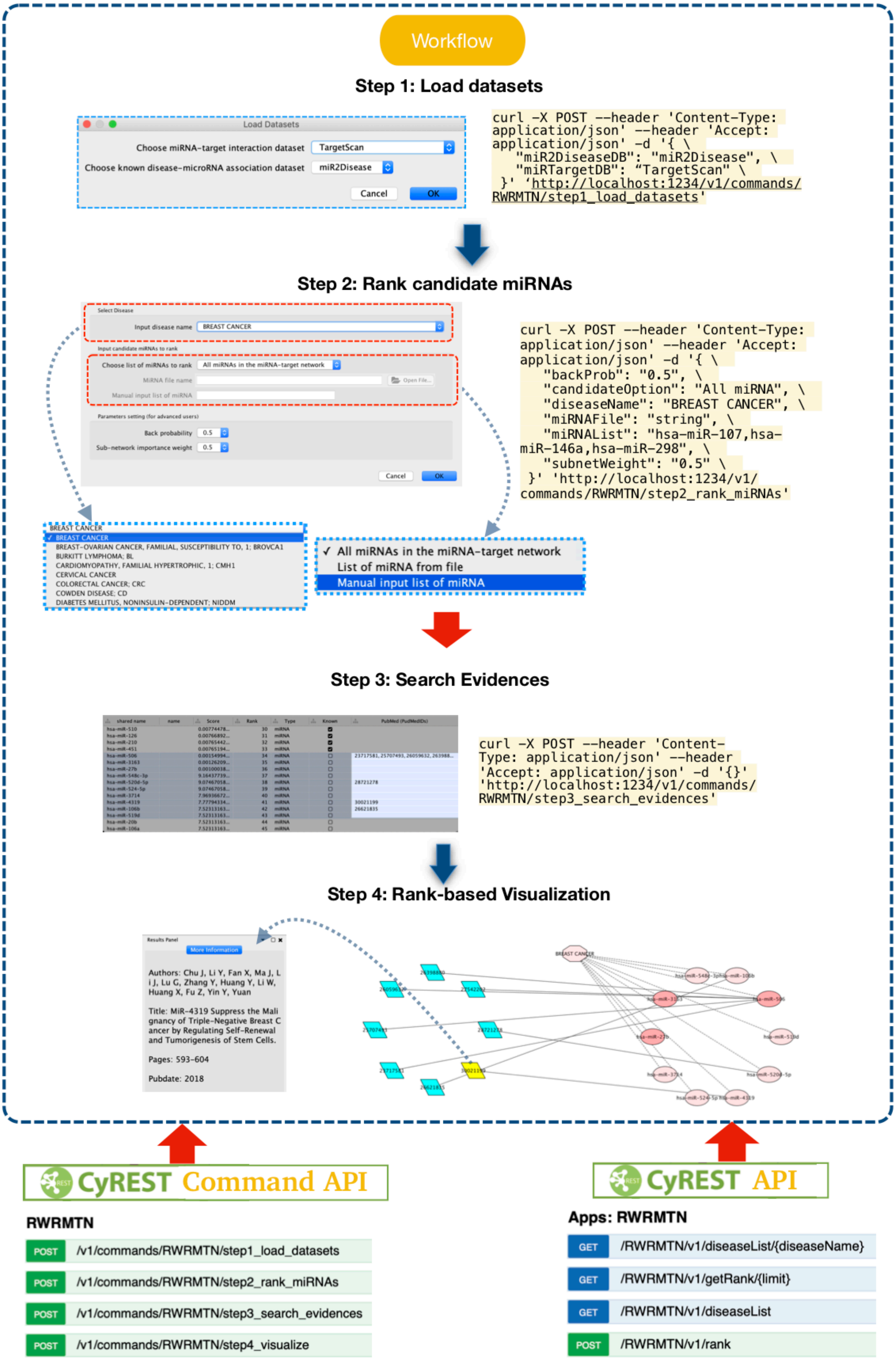

1. Run RWRMTN using Cytoscape menu and CyREST Command API

Step 1: Load datasets

Load datasets for miRNA-target interaction network and known disease-miRNA associations:

- miRNA-target gene interaction dataset (*miRTargetDB*): choose built-in dataset **TargetScan or miRWalk** or your own dataset to build miRNA-target interaction network.
- Known disease-miRNA association dataset (*miR2DiseaseDB*): choose built-in dataset **miR2Disease** or **HMDD**.

Step 1 can be performed by two ways:

- **Cytoscape menu: Apps →RWRMTN→ Step 1: Load datasets**

Here, a miRNA-target interaction dataset TargetScan (Lewis, et al., 2003) and a known disease-miRNA association dataset miR2Disease (Jiang, et al., 2009) were used.

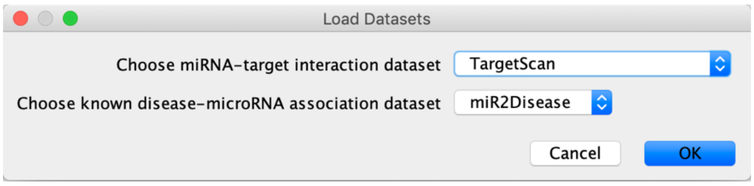

To build miRNA-target interaction network and load known disease-miRNA associations:

1. *Choose miRNA-target interaction dataset.*
2. *Choose known disease-microRNA association dataset*
3. *Click **OK** to load datasets.*

- **CyREST command API: Help → Automation → CyREST Command API.**

Choose RWRMTN in the SwaggerUI.

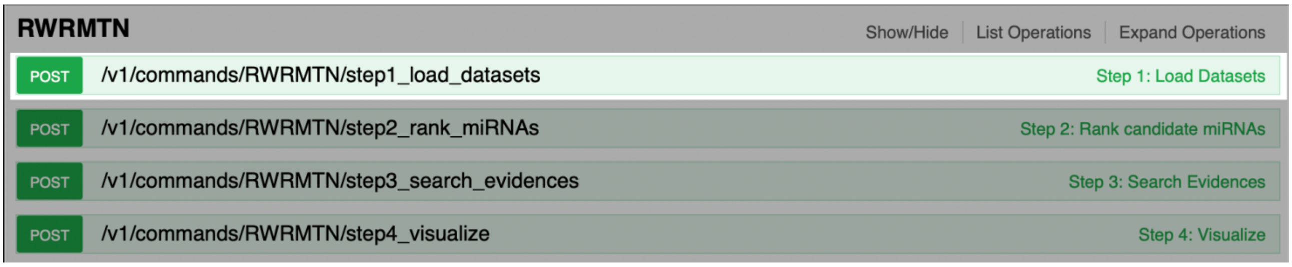

Fill the parameter requirement in the Parameter box and hit the button "Try it out"

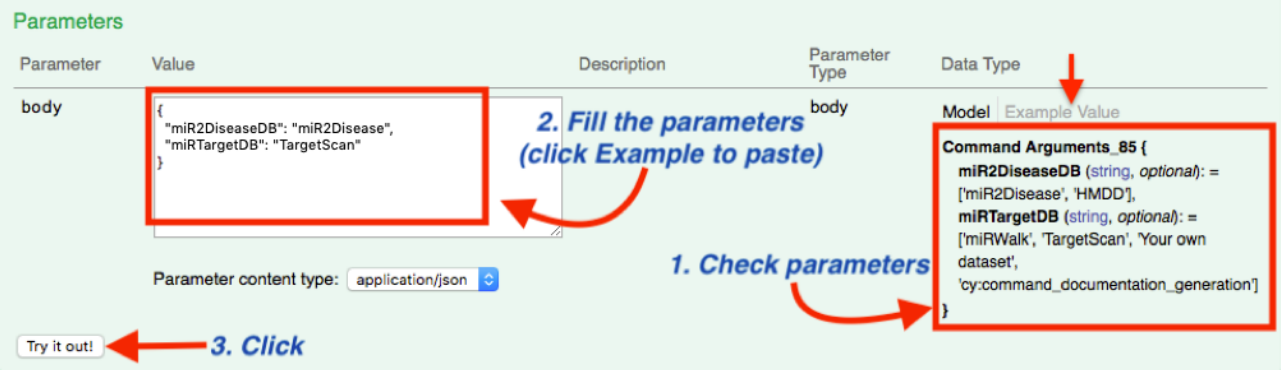

(For more details on using automation features (CyREST API and CyREST Command API) of Cytoscape, visit the site: https://github.com/cytoscape/cytoscape-automation/wiki/App-Developers:-Cytoscape-Command-Best-Practices)

- *This will load corresponding datasets into Network tab of Cytoscape (see following figure).*

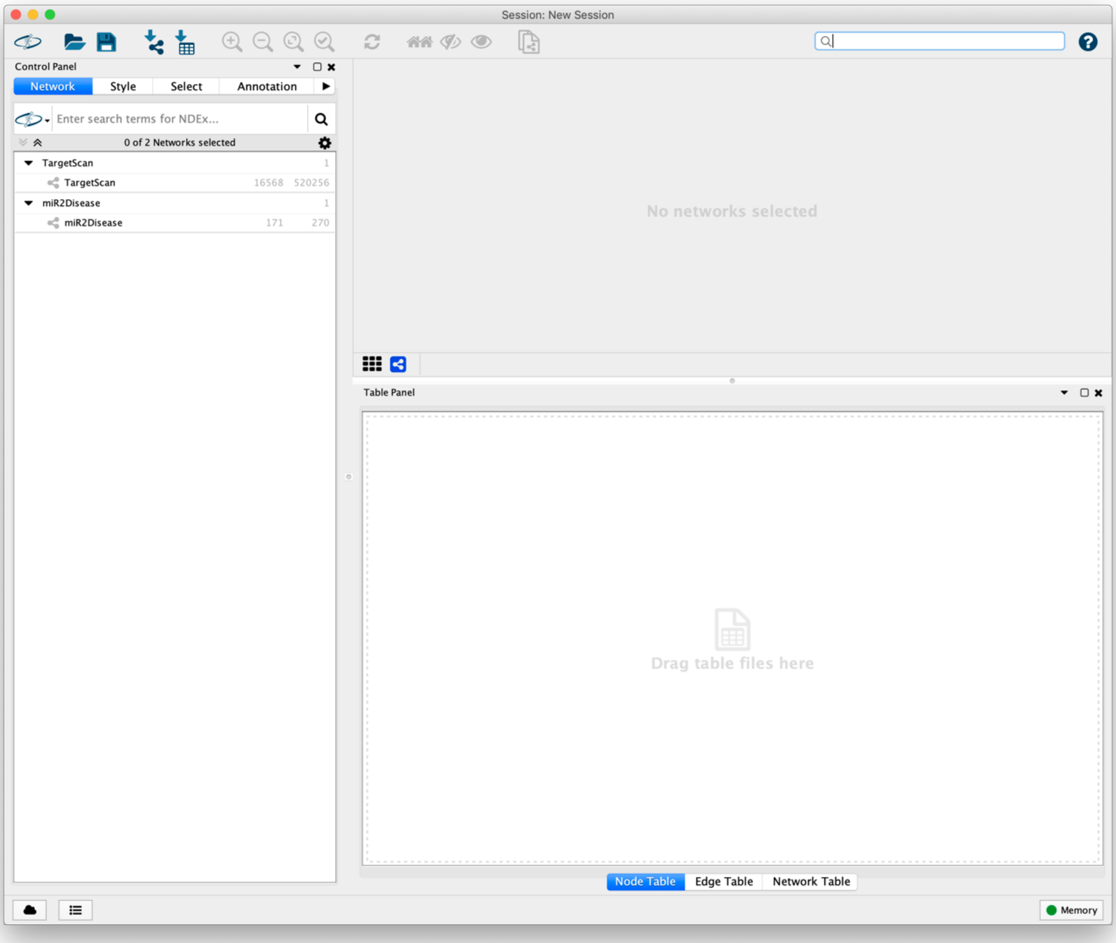

Note: a miRNA-target interaction dataset TargetScan (Lewis, et al., 2003) and a known disease-miRNA association dataset miR2Disease (Jiang, et al., 2009) were used.

For CyREST command API, return successful message:

Response Body


```
{
  "data": {
    "message": "Load Heterogeneous Network successfully"
  },
  "errors": []
}
```


Note that:

- *For miRNA-target datasets: We pre-installed 2databases **miRWalk** (database of experimentally validated miRNA-target interactions) and **TargetScan** (a dataset containing predicted miRNA-target interactions). If you want to use your own dataset, it must follow the following format, and be imported into Cytoscape beforehand.*

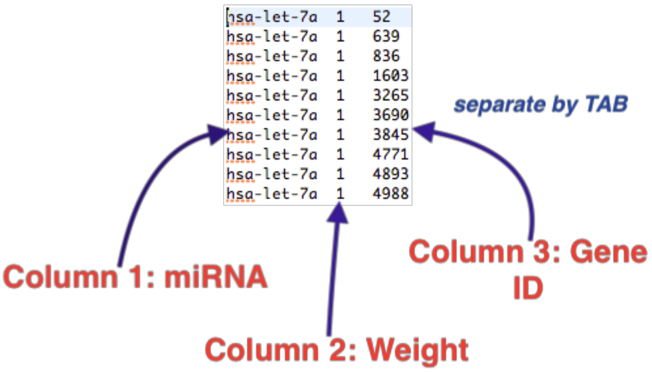

*Your own dataset can be imported into Cytoscape by clicking menu **File → Import → Network → File**. Set column 1 as source node and column 3 as target node.*

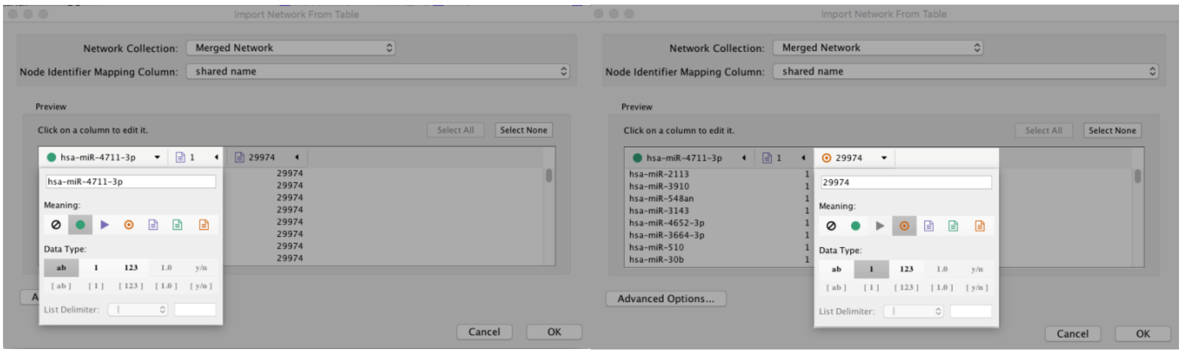

Then, it will appear in miRNA network option:

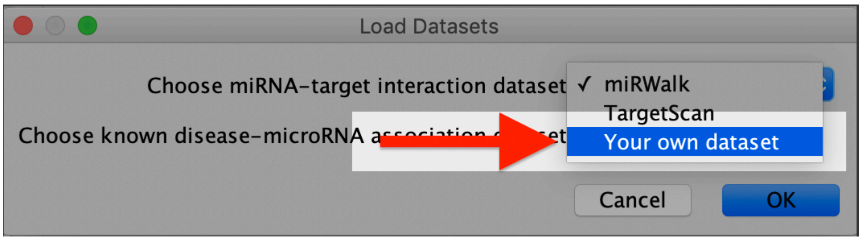

- *For known disease-miRNAs dataset: We pre-installed 2 datasets **miR2Disease** and **HMDD** (an up-to-date human disease-miRNA association database).*

<u>Step 2: Rank candidate miRNAs</u>

This step includes 4 sub-steps:

- Select a disease: For example, Breast cancer (OMIM ID: 114480) is selected.
- Input candidate miRNAs to rank: there are 3 options to choose
  - All miRNAs in the miRNA-target network
  - List of miRNAs from file: input the file.
  - Manual input list of miRNAs.
- Parameters setting (for advanced users)
  - Back probability (default setting is 0.5)
  - Sub-network importance weight (default setting is 0.5)
- Rank

Step 2 can be performed by two ways:

Cytoscape menu: Apps →RWRMTN→ Step 2: Rank candidate miRNAs

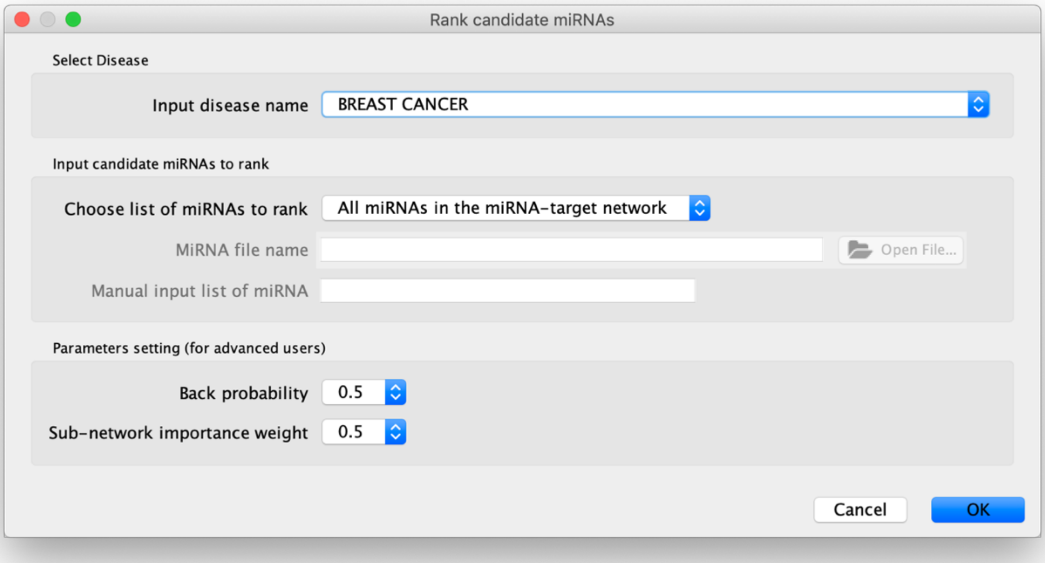

- ***Step 2.1***: Choose Disease name from the dropdown list:

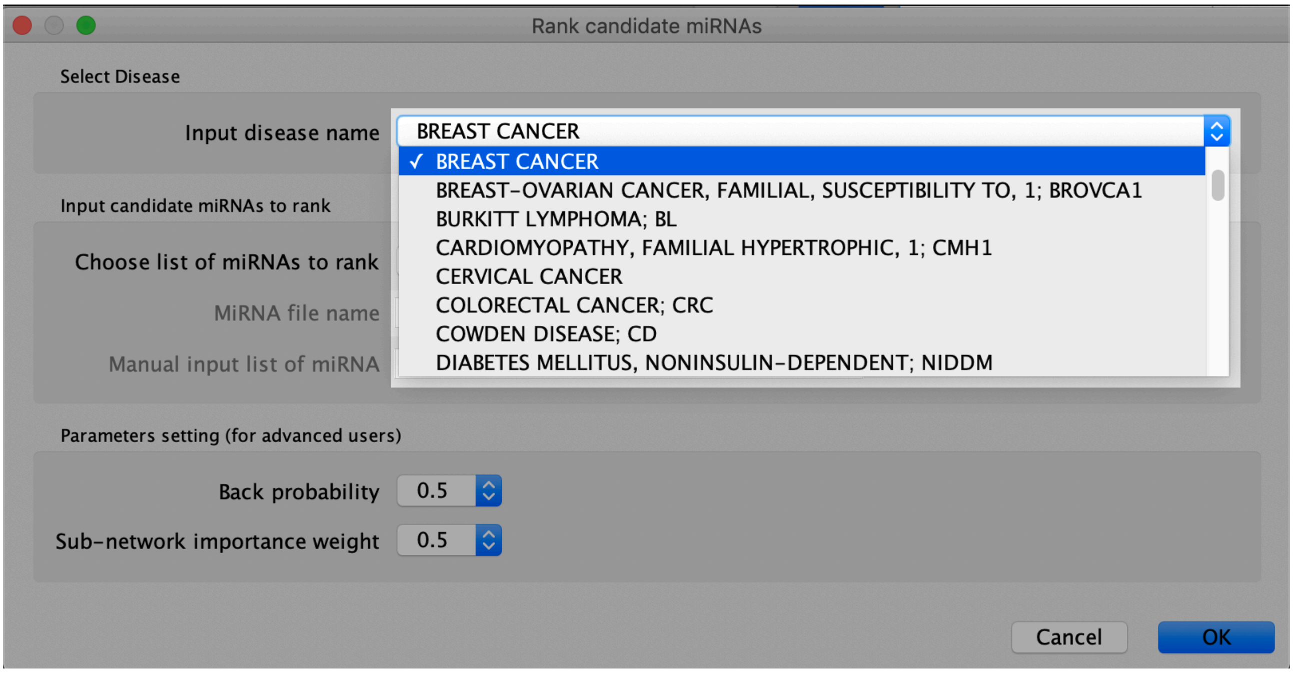

- ***Step 2.2***: Input candidate miRNAs to rank:
  Default setting is "All miRNAs in the network". If you want to choose specific list of miRNAs, you can select other options.

Select a file containing candidate miRNAs to be ranked.

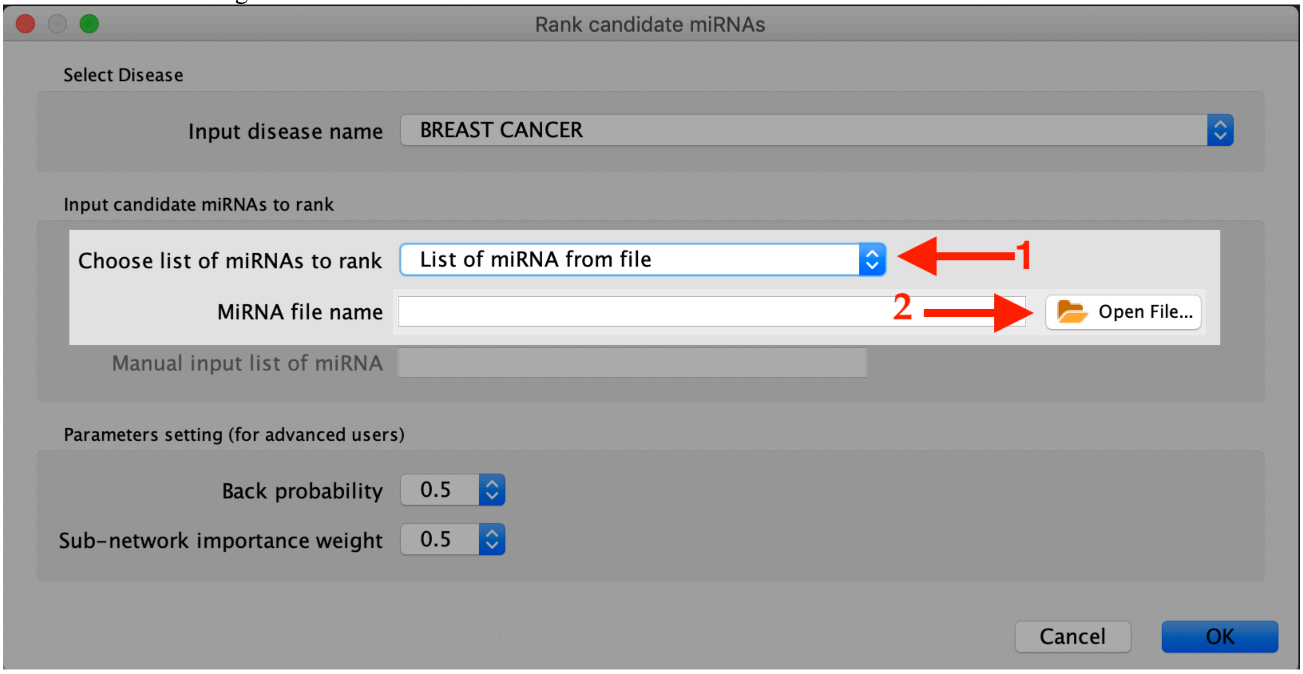

Directly input list of candidate miRNAs

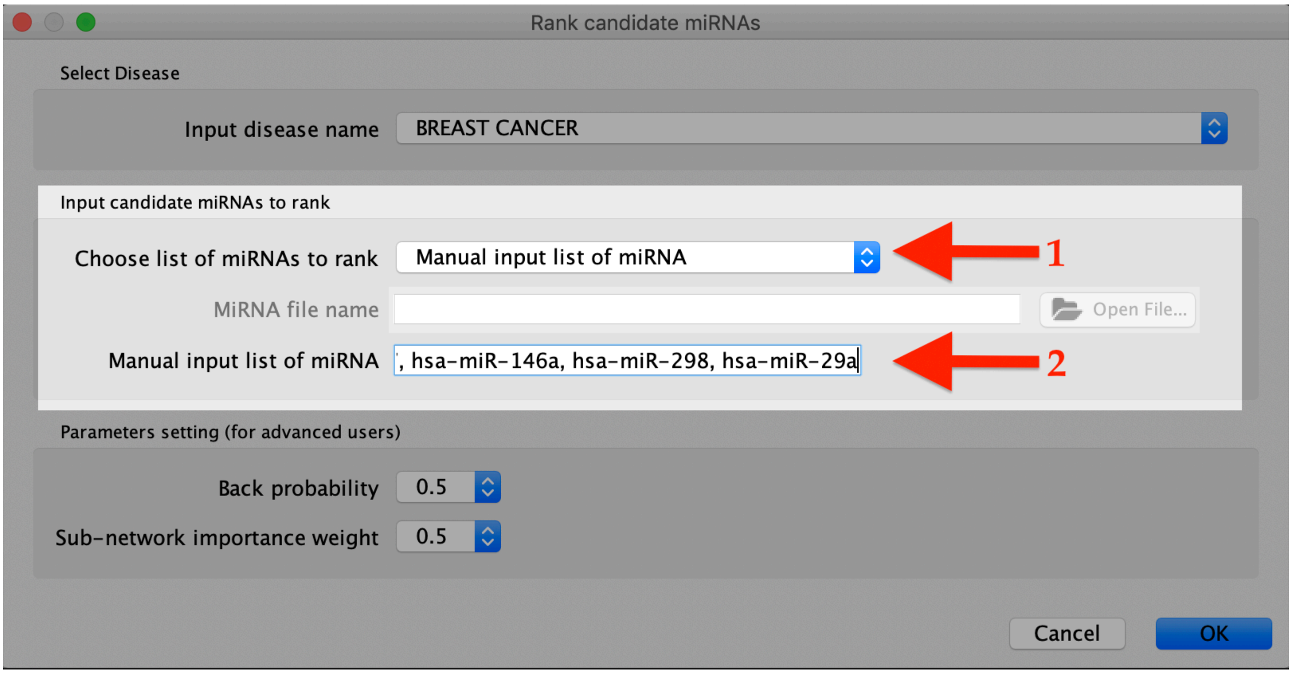

- *__Step 2.3__*: Parameters setting (for advanced users)

Choose to set Back probability and Sub-network importance weight in case of using different values other than default values.

- *__Step 2.4__*: Rank (Click **OK**)

- **CyREST command API: Help → Automation → CyREST Command API**

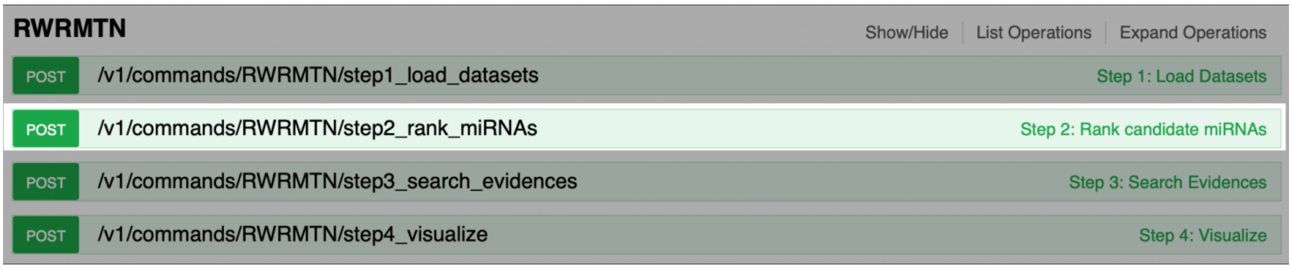

Fill all the parameter requirement in the Parameter box and hit the button "Try it out"

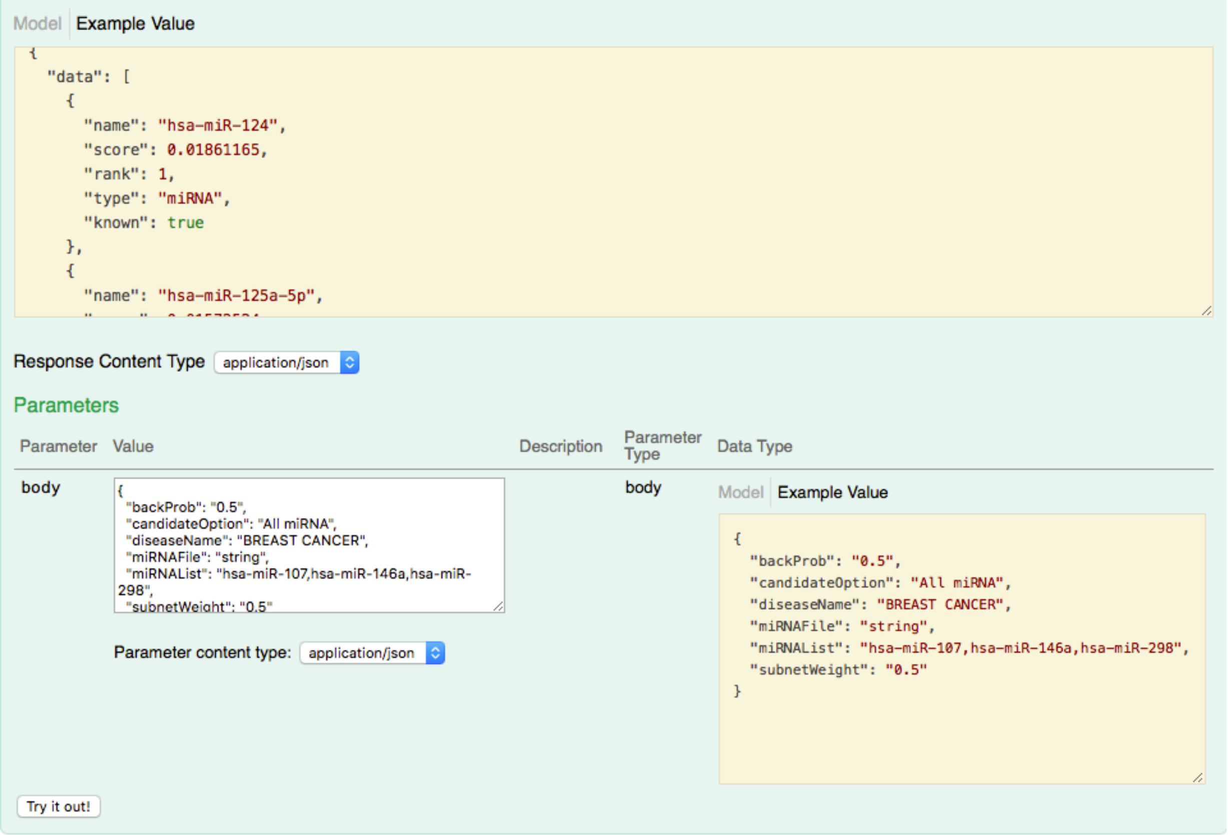

Step 2 will generate list of ranked miRNAs displayed in a network named by the selected disease (i.e. BREAST CANCER)

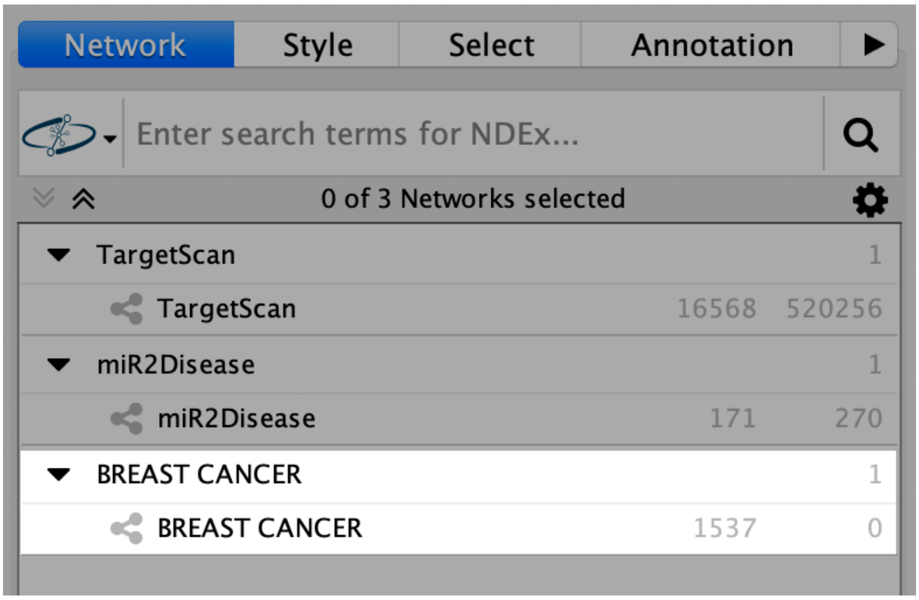

The following table lists known miRNAs associated with the selected diseases and the inputted candidate miRNAs

shared name	name	Score	Rank	Type	Known
hsa-miR-124		0.00912531...	1	miRNA	☑
hsa-miR-27a		0.00857614...	2	miRNA	☑
hsa-miR-128		0.00840957...	3	miRNA	☑
hsa-miR-200c		0.00838510...	4	miRNA	☑
hsa-miR-200b		0.00838510...	5	miRNA	☑
hsa-miR-429		0.00838123...	6	miRNA	☑
hsa-miR-125b		0.00835355...	7	miRNA	☑
hsa-miR-125a-5p		0.00835355...	8	miRNA	☑
hsa-miR-17		0.00832807...	9	miRNA	☑
hsa-miR-20a		0.00832807...	10	miRNA	☑
hsa-let-7a		0.00826798...	11	miRNA	☑
hsa-miR-206		0.00818675...	12	miRNA	☑
hsa-miR-200a		0.00817063...	13	miRNA	☑
hsa-miR-141		0.00817063...	14	miRNA	☑
hsa-miR-204		0.00814230...	15	miRNA	☑
hsa-miR-373		0.00805362...	16	miRNA	☑
hsa-miR-516a-3p		0.00803928...	17	miRNA	☑
hsa-miR-155		0.00802905...	18	miRNA	☑
hsa-miR-7		0.00794792...	19	miRNA	☑
hsa-miR-205		0.00794064...	20	miRNA	☑
hsa-miR-221		0.00790413...	21	miRNA	☑
hsa-miR-222		0.00790413...	22	miRNA	☑
hsa-miR-127-5p		0.00790080...	23	miRNA	☑
hsa-miR-335		0.00786437...	24	miRNA	☑
hsa-miR-146a		0.00784430...	25	miRNA	☑
hsa-miR-146b-5p		0.00784430...	26	miRNA	☑
hsa-miR-21		0.00781843...	27	miRNA	☑
hsa-miR-520c-5p		0.00778787...	28	miRNA	☑
hsa-miR-10b		0.00778371...	29	miRNA	☑
hsa-miR-510		0.00774478...	30	miRNA	☑
hsa-miR-126		0.00766892...	31	miRNA	☑
hsa-miR-210		0.00765442...	32	miRNA	☑
hsa-miR-451		0.00765194...	33	miRNA	☑
hsa-miR-506		0.00154994...	34	miRNA	☐
hsa-miR-3163		0.00126209...	35	miRNA	☐
hsa-miR-27b		0.00100038...	36	miRNA	☐
hsa-miR-548c-3p		9.16437739...	37	miRNA	☐
hsa-miR-520d-5p		9.07467058...	38	miRNA	☐
hsa-miR-524-5p		9.07467058...	39	miRNA	☐
hsa-miR-3714		7.96936672...	40	miRNA	☐
hsa-miR-4319		7.77794334...	41	miRNA	☐
hsa-miR-106b		7.52313163...	42	miRNA	☐
hsa-miR-519d		7.52313163...	43	miRNA	☐
[illegible]		[illegible]	[illegible]	[illegible]	[illegible]

And JSON result is exposed in CyREST Command API

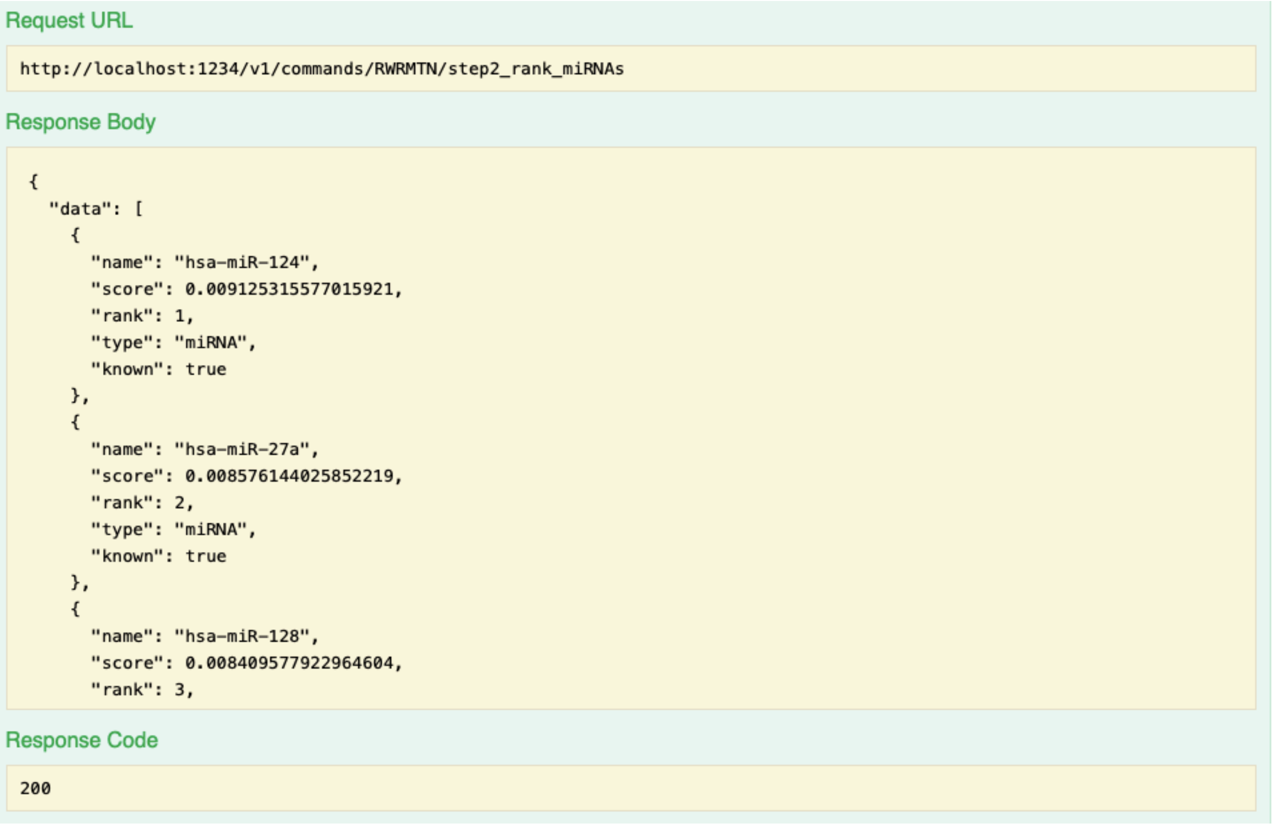

<u>Step 3: Search Evidences</u>

By selecting option "All miRNAs in the miRNA-target network" in Step 2, all miRNAs in the selected miRNA-target gene network are ranked and displayed in Node Table of the network (in Network Tab of Cytoscape) which has the same name as the disease of interest (i.e., BREAST CANCER).

To find the evidences in literature (PubMed), you need to select highly ranked miRNAs by highlighting rows in the network (i.e., BREAST CANCER)and choose between these two ways:

- **Cytoscape menu**: Select menu **Apps → RWRMTN → Step 3: Search Evidences**
- **CyREST command API: Help → Automation → CyREST Command API.**

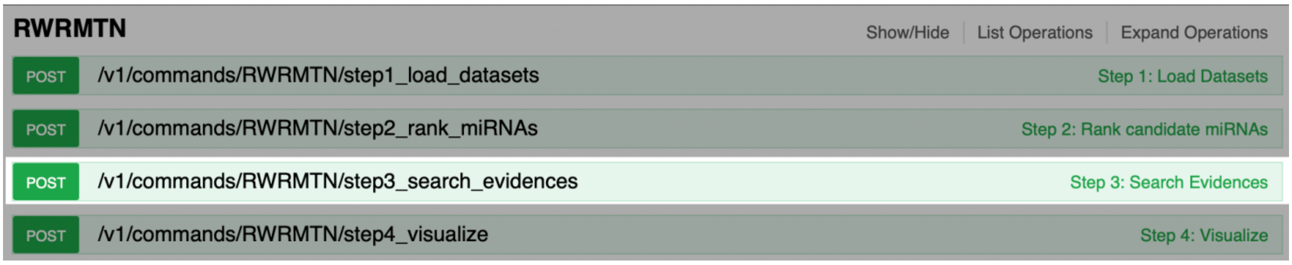

Hit the button "Try it out" without any parameters (remember to highlight rows first).

<u>Note</u>*: If you receive the alert that none row is selected. You need to show the column "selected" in Cytoscape table and set value to* ***true*** *once to trigger the function "selected" column to work.*

The result of Step 3 is a list of PubMed IDs of the publications containing evidences about associations between selected miRNAs and the disease of interest displayed in . For more information (e.g., paper title, author list, publication date, etc..), refer to Step 4 or use command API of step 3.

shared name	name	Score	Rank	Type	Known	PubMed (PudMedIDs)
hsa-miR-510		0.00774478...	30	miRNA	☑	
hsa-miR-126		0.00766892...	31	miRNA	☑	
hsa-miR-210		0.00765442...	32	miRNA	☑	
hsa-miR-451		0.00765194...	33	miRNA	☑	
hsa-miR-506		0.00154994...	34	miRNA	☐	23717581, 25707493, 26059632, 263988...
hsa-miR-3163		0.00126209...	35	miRNA	☐	
hsa-miR-27b		0.00100038...	36	miRNA	☐	
hsa-miR-548c-3p		9.16437739...	37	miRNA	☐	
hsa-miR-520d-5p		9.07467058...	38	miRNA	☐	28721278
hsa-miR-524-5p		9.07467058...	39	miRNA	☐	
hsa-miR-3714		7.96936672...	40	miRNA	☐	
hsa-miR-4319		7.77794334...	41	miRNA	☐	30021199
hsa-miR-106b		7.52313163...	42	miRNA	☐	26621835
hsa-miR-519d		7.52313163...	43	miRNA	☐	
hsa-miR-20b		7.52313163...	44	miRNA	☐	
hsa-miR-106a		7.52313163...	45	miRNA	☐	

This result is exposed by using CyREST Command API

Request URL

```
http://localhost:1234/v1/commands/RWRMTN/step3_search_evidences
```

Response Body


```
{
  "data": [
    {
      "miRnaName": "hsa-miR-520d-5p",
      "PubMedIds": [
        "28721278"
      ],
      "info": {
        "28721278": {
          "pubdate": "2016",
          "authors": [
            "Ishihara Y",
            "Tsuno S",
            "Ping B",
            "Ashizaki T",
            "Nakashima M",
            "Miura K",
            "Miura Y",
            "Yamashita T",
            "Hasegawa J",
```


Response Code

```
200
```

As can be seen from the above screenshot, the result is returned as an array of JSON objects. Each object includes 3 pairs of key/value:

- "miRnaName" is the selected miRNA.
- "PubMedIds" lists the ids found in the PubMed which provided evidences of associations between selected miRNA and the disease of interest.
- "info" shows the detail information of each PubMed ID including publication date, authors, title and pages, which then can be seen by visualization functions of RWRMTN.

In this case study, four of ten highly ranked miRNAs are provided with evidences.

- "hsa-miR-506" supported by five studies (PubMed IDs: 23717581, 25707493, 26059632, 26398880 and 27542202). The study 23717581 showed that has-miR-506 regulates epithelial mesenchymal transition in breast cancer cell lines. Meanwhile, the study 26059632 proved notable inhibition of hsa-miR-506 over-expression to proliferation and metastasis of breast cancer cells. In addition, study 26398880 indicated that mechanism underlying miRNA-506 is a contributing factor in breast carcinogenesis (has-miR-506 was proven to be a tumor suppressor).
- "hsa-miR-520d-5p" supported by a study with PubMed ID 28721278. More specifically, it was reported that this miRNA upregulates the activation of BRCA1 (breast cancer 1, early onset) in the DNA repair process – 35 days after transfection.
- "hsa-miR-4319" was showed in study PubMed ID 30021199 as a suppressor of the malignancy of triple-negative breast cancer by regulating self-renewal and tumorigenesis of stem cells.
- "has-miR-106b" was proven by the experiment carried on patient samples and cell lines in the study (PubMed ID 26621835)

Step 4: Visualize

Selected miRNAs can be visualized in a network based on the rankings. In addition, target genes, the disease of interest and detail information of PubMed IDs collected from Step 3 such as *paper title, author list, journal name* can be displayed aside in this network.

Choose between two options for visualization ***(Remember to highlight selected rows first)***

- **Cytoscape menu:** Select menu **Apps → RWRMTN → Step 4: Visualize**

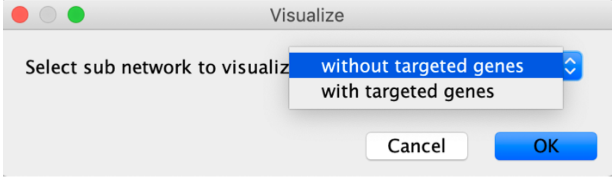

Visualization options:

1. *Visualize* ***without*** *targeted genes of selected miRNAs*
2. *Visualize* ***with*** *targeted genes of selected miRNAs*
3. *Click* ***OK*** *to visualize.*

- **CyREST command API: Help → Automation → CyREST Command API**

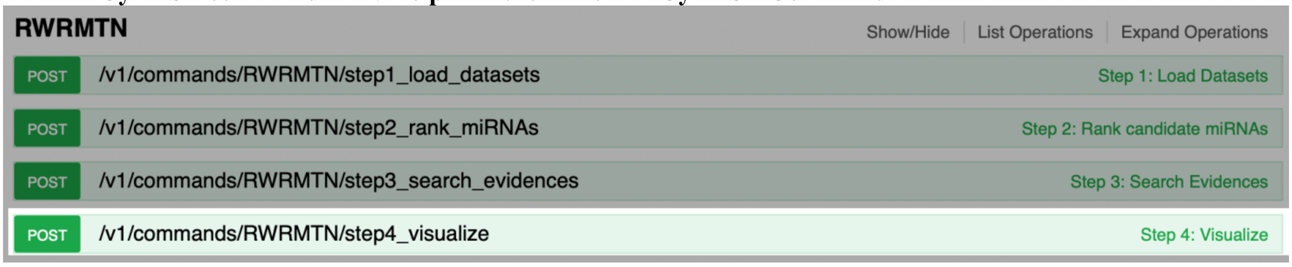

Fill the parameter requirement in the Parameter box and hit the button "Try it out"

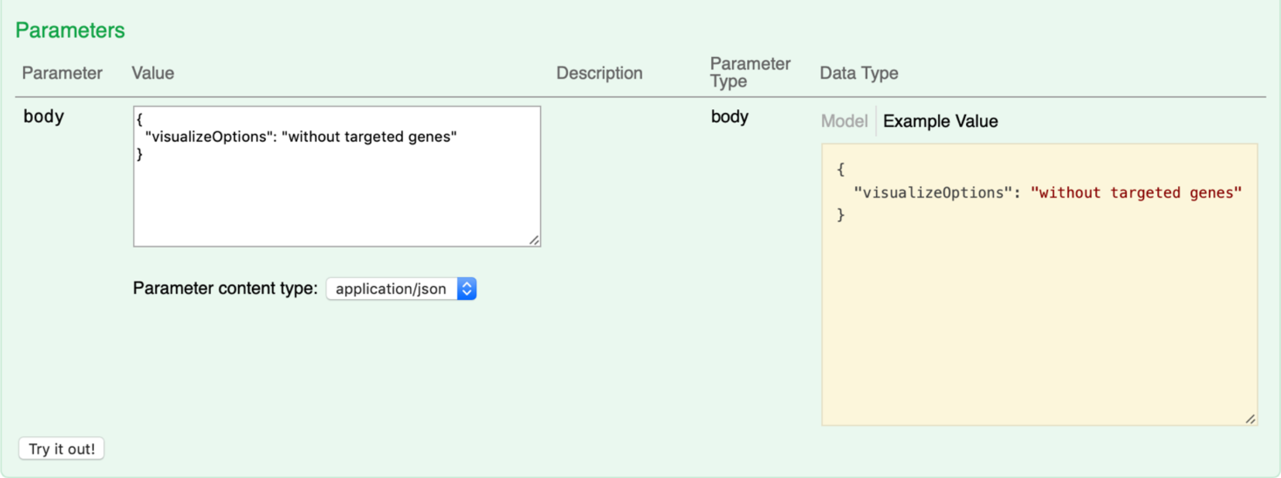

Step 4 will create network view in two cases:

- *Without targeted genes*

Here, we selected top 10 ranked candidate miRNAs and then visualized based on their rankings. Rank difference is reflected by red color's tone. The higher rank is represented by darker red. In addition, evidences (PubMed IDs) for each selected miRNAs were also visualized. Disease and candidate miRNAs are connected by long-dash lines, meanwhile solid lines are used to connect PubMed IDs and miRNAs.

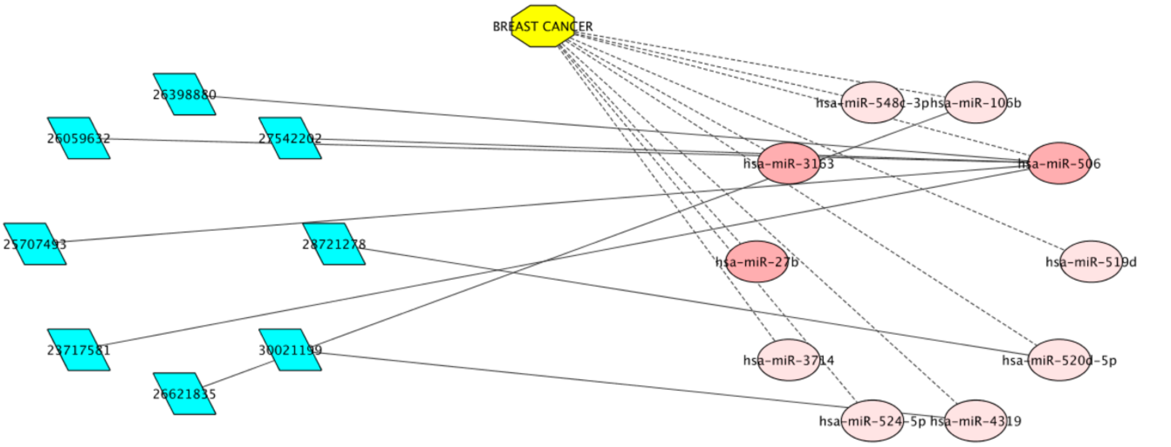

(*Note:* PubMed IDs, disease, and miRNAs are represented in parallelogram, octagon and ellipse shapes, respectively)

One may want to view the relationships with miRNAs known to be associated with the disease of interest (i.e., BREAST CANCER). Thus, we can additionally select known miRNAs to visualize

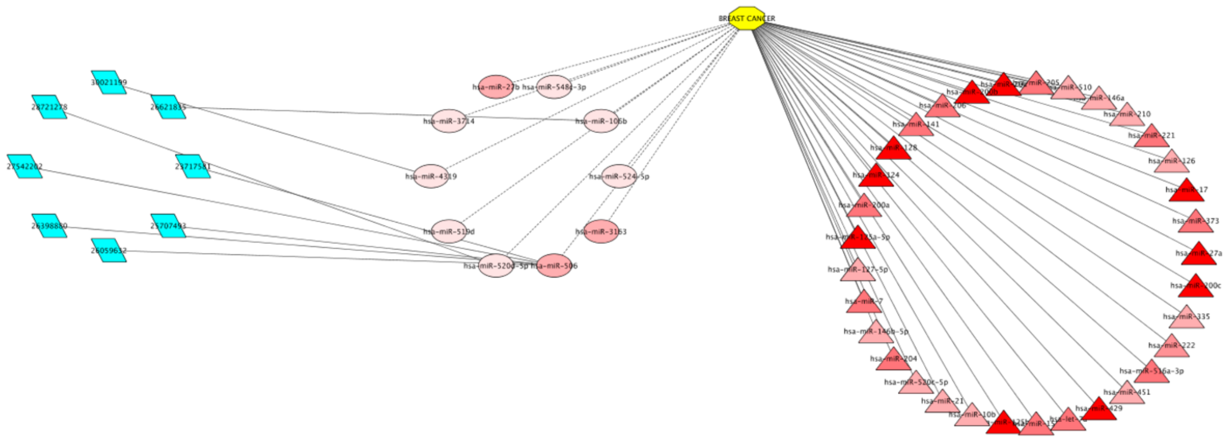

(*Note:* Known miRNAs are represented by triangles which are connected with the disease of interest by dot lines).

For detail information of each PubMed ID, right click on PubMed ID node and select menu **RWRMTN → Check Information**

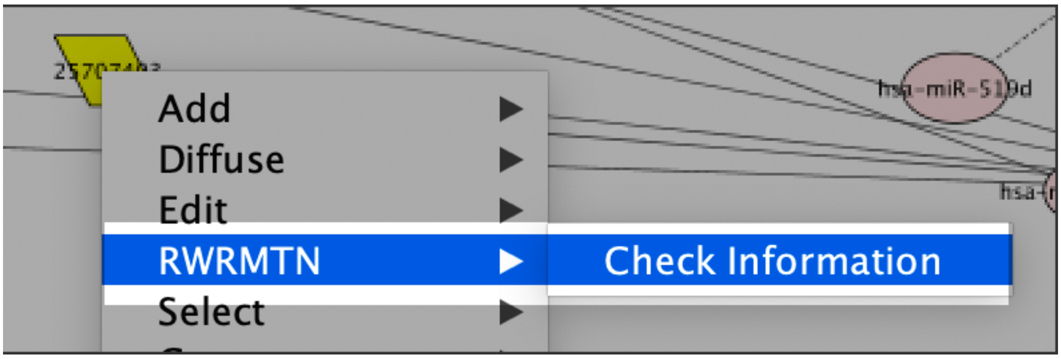

The East panel will appear to display detail information of the selected PubMed ID.

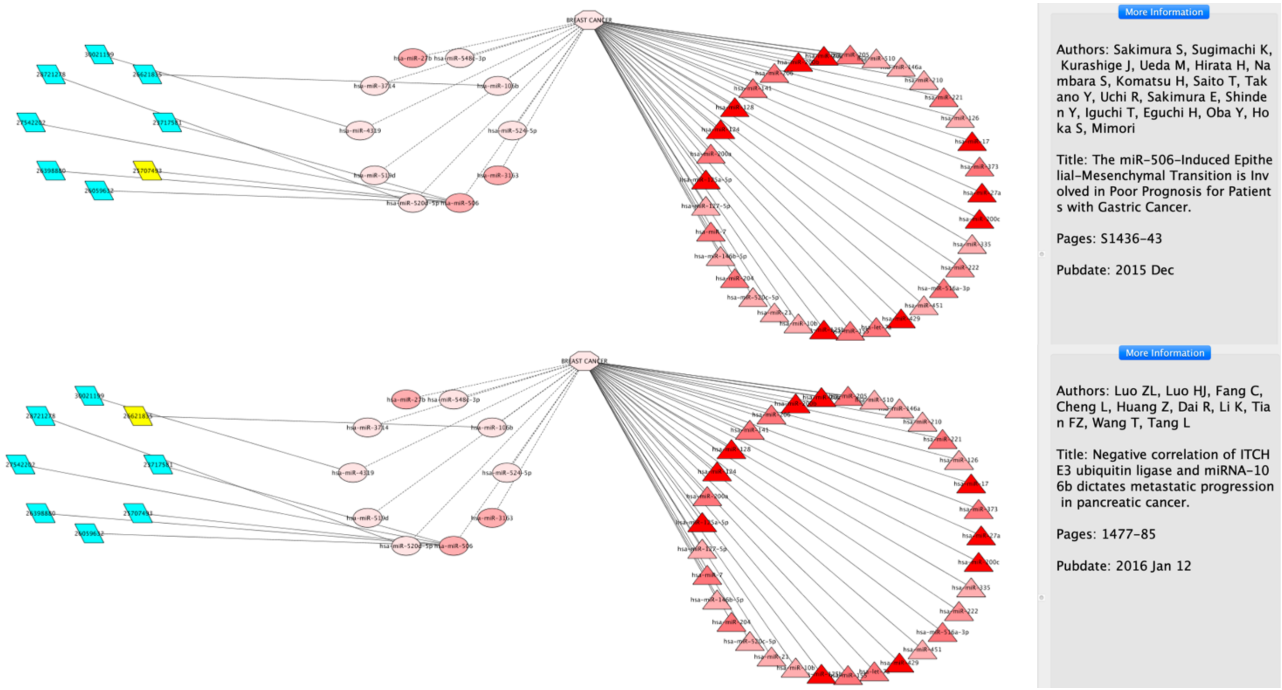

- *With targeted genes*

Select this option if user wants to see the relationships between the selected miRNAs and their target genes.

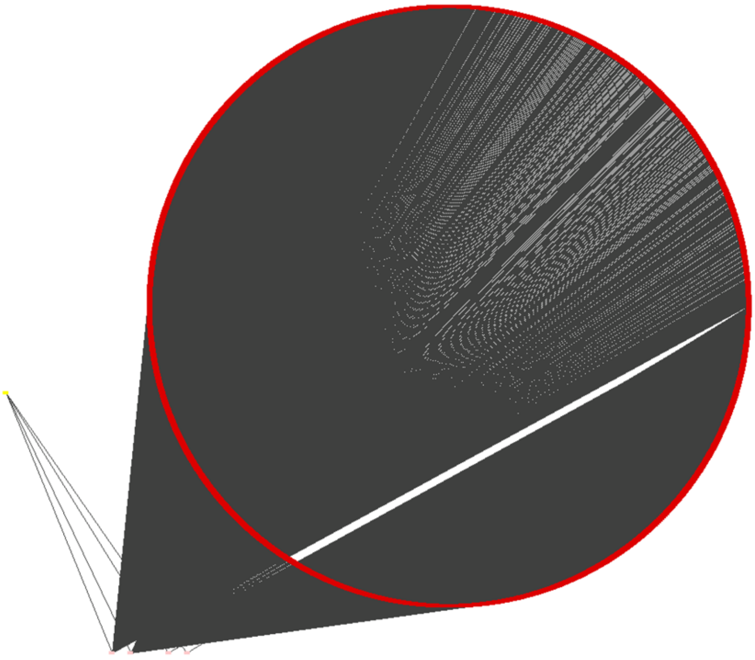

(Note: Each miRNA often targets to many genes)

2. Run RWRMTN by calling CyREST API

In this section, we first introduce some developed CyREST APIs which provides some helpful functions. Second, we demonstrate of their use in a workflow in R statistic environment.

Overview of CyREST APIs

To reveal all APIs, select menu **Help → Automation → CyREST API** to open Swagger UI of CyREST API**.** Here is the list of CyREST APIs of RWRMTN.

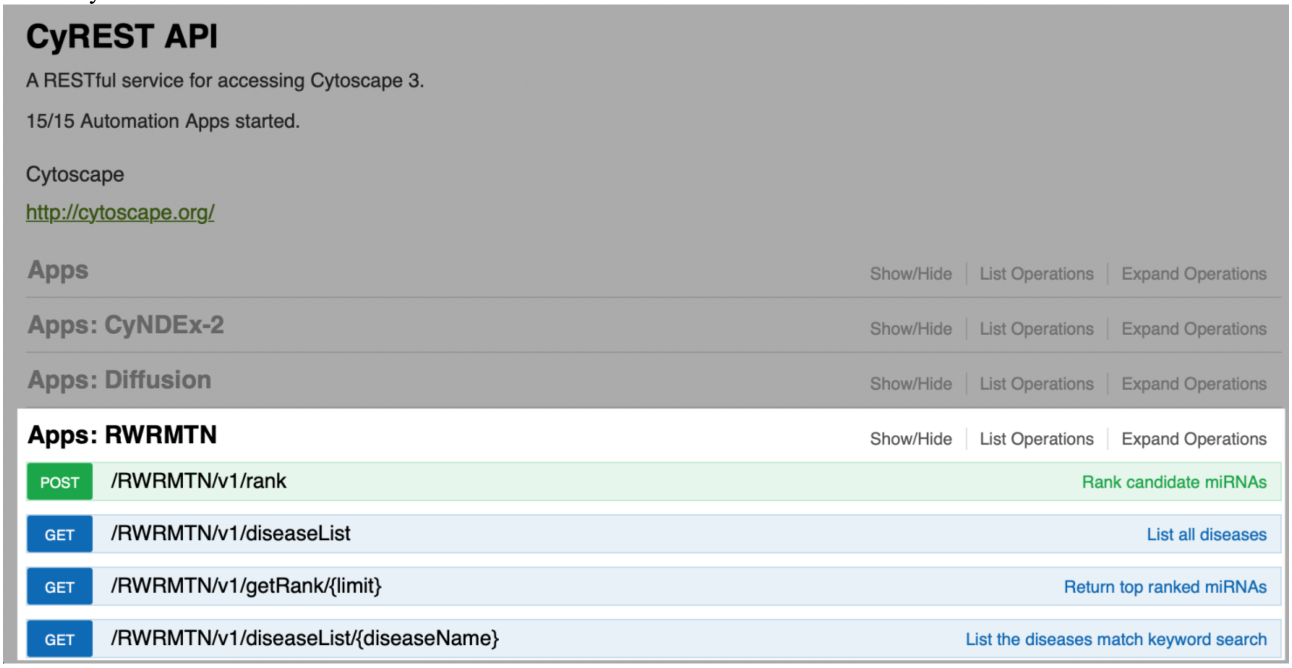

The detail of each API is available in Swagger Documentation. Here is short description.

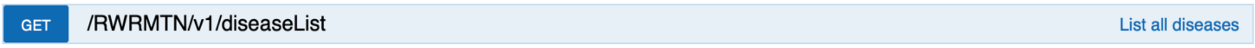

This API returns list of all diseases (OMIM ID and disease name) available in the selected known disease-miRNA association database (i.e., miR2Disease). Based on this list, users can select a disease of interest.

Request URL

```
http://localhost:1234/RWRMTN/v1/diseaseList
```

Response Body


```
[
  {
    "diseaseID": "MIM104300",
    "diseaseName": "ALZHEIMER DISEASE; AD"
  },
  {
    "diseaseID": "MIM109800",
    "diseaseName": "BLADDER CANCER"
  },
  {
    "diseaseID": "MIM113970",
    "diseaseName": "BURKITT LYMPHOMA; BL"
  },
  {
    "diseaseID": "MIM114480",
    "diseaseName": "BREAST CANCER"
  },
  {
    "diseaseID": "MIM114500",
    "diseaseName": "COLORECTAL CANCER; CRC"
```


GET /RWRMTN/v1/diseaseList/{diseaseName} List the diseases match keyword search

This API provides a list of diseases whose names match the query parameter (e.g., cancer). This API help user narrow down list of diseases to the disease of interest (e.g., disease ID MIM114480 for BREAST CANCER).
For example:

Request URL

```
http://localhost:1234/RWRMTN/v1/diseaseList/cancer
```

Response Body


```
[
  {
    "diseaseID": "MIM109800",
    "diseaseName": "BLADDER CANCER"
  },
  {
    "diseaseID": "MIM114480",
    "diseaseName": "BREAST CANCER"
  },
  {
    "diseaseID": "MIM114500",
    "diseaseName": "COLORECTAL CANCER; CRC"
  },
  {
    "diseaseID": "MIM133239",
    "diseaseName": "ESOPHAGEAL CANCER"
  },
  {
    "diseaseID": "MIM137215",
    "diseaseName": "GASTRIC CANCER, HEREDITARY DIFFUSE; HDGC"
```


POST /RWRMTN/v1/rank Rank candidate miRNAs

This API will use RWRMTN to rank candidate miRNAs and return the result in JSON format. The request is POST request with input parameters as follows:

required:
Disease OMIM ID: String
List of miRNA: String

optional:
miRTargetDB: String
miR2DiseaseDB: String
backProb: String
subnetWeight: String

Model | Example Value

RankParameters {
diseaseOMIMID (string): Disease OMIM ID,
listOfmiRNAs (string): List of miRNA to rank,
miRTargetDB (string, *optional*): MicroRNA Dataset,
miR2DiseaseDB (string, *optional*): Disease-miRNA Dataset,
backProb (number, *optional*): Back-probability,
subnetWeight (number, *optional*): Sub-network importance weight
}

Model | Example Value


```
{
  "diseaseOMIMID": "MIM114480",
  "listOfmiRNAs": "All miRNAs",
  "miRTargetDB": "TargetScan",
  "miR2DiseaseDB": "miR2Disease",
  "backProb": 0.5,
  "subnetWeight": 0.5
}
```


The result is:

Request URL

```
http://localhost:1234/RWRMTN/v1/rank
```

Response Body


```
[
  {
    "rnaName": "hsa-miR-124",
    "rnaScore": 0.009125315577015921,
    "rnaRank": 1,
    "type": "miRNA",
    "known": true
  },
  {
    "rnaName": "hsa-miR-27a",
    "rnaScore": 0.008576144025852219,
    "rnaRank": 2,
    "type": "miRNA",
    "known": true
  },
  {
    "rnaName": "hsa-miR-128",
    "rnaScore": 0.008409577922964604,
    "rnaRank": 3,
    "type": "miRNA",
```


Response Code

```
200
```

GET /RWRMTN/v1/getRank/{limit} — Return top ranked miRNAs

This API returns top ranked miRNAs by setting {limit} parameter.

For example: The following query returns top 10 ranked miRNAs

Request URL

```
http://localhost:1234/RWRMTN/v1/getRank/10
```

Response Body


```
[
  {
    "rnaName": "hsa-miR-124",
    "rnaScore": 0.009125315577015921,
    "rnaRank": 1,
    "type": "miRNA",
    "known": true
  },
  {
    "rnaName": "hsa-miR-27a",
    "rnaScore": 0.008576144025852219,
    "rnaRank": 2,
    "type": "miRNA",
    "known": true
  },
  {
    "rnaName": "hsa-miR-128",
    "rnaScore": 0.008409577922964604,
    "rnaRank": 3,
    "type": "miRNA",
```


Response Code

```
200
```

Using RWRMTN in a workflow in R environment

In this case study, we used a dataset GSE19783 from GEO (Enerly, et al., 2011), which was created using Agilent-019118 Human miRNA Microarray 2.0 G4470B platform (GPL8227) and Agilent-014850 Whole Human Genome Microarray 4x44K G4112F (GPL6480) (https://www.ncbi.nlm.nih.gov/geo/query/acc.cgi?acc=GSE19783). The study characterizes breast cancer subtypes from joint analysis of high throughput miRNA (using GPL8227) and mRNA (using GPL6480) Data.
In this case study, we explored the 799 miRNAs that were differentially expressed between the 64 wild-type samples (WT) and 36 *TP53* mutant samples via a workflow in R environment using CyREST API.

Briefly, here is the workflow:

1. Download the datasets
2. Perform differential expression analysis with *limma* package
3. Select a list of miRNAs, whose differential expression between cases and controls is statistically significant, as candidates
4. Rank the candidate miRNAs by RWRMTN via a CyREST API using a miRNA-target interaction dataset miRWalk (Dweep, et al., 2011) and a known disease-miRNA association dataset HMDD (Li, et al., 2014). Note that CyREST API is hosted by Cytoscape platform so you need to open Cytoscape with installed RWRMTN (just open – not need to use GUI of RWRMTN).

Before running the analysis, make sure RWRMTN and necessary packages are installed and they are functional:

- Please run *Check_CytoscapeConnection_LibraryInstallation_RWRMTN.R* in Case Study folder (download at https://github.com/hauldhut/RWRMTN) for checking connection with Cytoscape and whether necessary packages are installed.

Then, run the following source code in R (the source code can be found in R_callCyRestAPI.R in Case Study folder (download at https://github.com/hauldhut/RWRMTN)

```
####################################################################
library(Biobase)
library(GEOquery)
library(limma)
library(httr)
library(jsonlite)

library(httr)
```

```r
library(jsonlite)

### Load series and platform data from GEO
gset <- getGEO("GSE19783", GSEMatrix =TRUE, AnnotGPL=FALSE)
if (length(gset) > 1){
  idx <- grep("GPL8227", attr(gset, "names"))
}else{
  idx <- 1
}

gset <- gset[[idx]]

# make proper column names to match toptable
fvarLabels(gset) <- make.names(fvarLabels(gset))

TP53Status<-gset$`tp53 mutation status:ch1`
# labeling for all samples
gsms<-""

sml<-c()
for(i in 1:length(TP53Status)){
  if(TP53Status[i]=="Mut"){
    gsms<-paste0(gsms,"1")
    sml[i]<-"Group1"
  }else{
    gsms<-paste0(gsms,"0")
    sml[i]<-"Group0"
  }
}

#Retrieve Expression Data From ESets
ex <- exprs(gset)
ex[which(ex <= 0)] <- NaN
# log2 transform
exprs(gset) <- log2(ex)

### Differential expression analysis with limma package
# set up the data and proceed with analysis
fl <- as.factor(sml)
gset$description <- fl
#creates a design (or model) matrix
design <- model.matrix(~ description + 0, gset)
colnames(design) <- levels(fl)
fit <- lmFit(gset, design)
cont.matrix <- makeContrasts(Group1-Group0, levels=design)
fit2 <- contrasts.fit(fit, cont.matrix)
fit2 <- eBayes(fit2, 0.01)
diffmiRNAlist <- topTable(fit2, adjust="fdr", number=nrow(fit2))

#Only select miRNAs whose differential expression between the two group (Group1 & Group0)
#is statistically significant  (adj.P.val <=0.05) for ranking with RWRMTN
sigmiRNAlist <- subset(diffmiRNAlist,adj.P.Val<=0.05) #This returns 85 miRNAs
sigmiRNAlist <- subset(sigmiRNAlist, select=c("ID","adj.P.Val","P.Value"))
colnames(sigmiRNAlist)<-c("rnaName", "adj.P.Val","P.Value")

#Save statistically significant miRNAs standard output
write.table(sigmiRNAlist, file=stdout(), row.names=F, sep="\t")


###Rank statistically significant miRNAs (candidate miRNAs) with RWRMTN
#Get miRNA list
lr<-sigmiRNAlist$rnaName
lor<-""
n<-length(lr)
for(i in 1:n){
  lor<-paste(lor, lr[i],", ",sep='')
}

#Select datasets (miRTargetDB, miR2DiseaseDB), the disease of interest (MIM114480: Breast cancer)
```

```
#and pass the candidata miRNAs list
login <- list(
  diseaseOMIMID= "MIM114480",#OMIM ID of Breast cancer
  listOfmiRNAs= lor,
  miRTargetDB= "miRWalk",
  miR2DiseaseDB= "HMDD"
)

#Run Cytosacpe CyREST API
request_body_json <- toJSON(login)
res <- POST("http://localhost:1234/RWRMTN/v1/rank", body = login, encode="json")
y<-httr::content(res,"text", encoding = 'UTF-8')
get_prices_json <- fromJSON(y, flatten = TRUE)

Output <- fromJSON((y))
#Remove miRNA with rank=0 (which are not available on the miRNA-target network)
rankedmiRNAlist <- Output[which(Output$rnaRank!=0),]
rankedmiRNAlist
write.csv(rankedmiRNAlist, file="rankedmiRNAlist.csv", row.names=F)
```

Here is the result:

```
           rnaName     rnaScore rnaRank  type known
1      hsa-miR-375 1.719388e-02       1 miRNA  TRUE
2      hsa-miR-107 4.059087e-03       2 miRNA  TRUE
3      hsa-miR-15a 3.420814e-03       3 miRNA  TRUE
4      hsa-miR-326 3.377178e-03       4 miRNA  TRUE
5      hsa-miR-155 2.825815e-03       5 miRNA  TRUE
6      hsa-miR-145 2.716448e-03       6 miRNA  TRUE
7     hsa-miR-148b 2.686323e-03       7 miRNA  TRUE
8      hsa-miR-224 2.610611e-03       8 miRNA  TRUE
9       hsa-let-7e 2.553925e-03       9 miRNA  TRUE
10     hsa-miR-29c 2.545947e-03      10 miRNA  TRUE
11     hsa-miR-26b 2.467229e-03      11 miRNA  TRUE
12     hsa-miR-30a 2.455210e-03      12 miRNA  TRUE
13      hsa-let-7b 2.428472e-03      13 miRNA  TRUE
14      hsa-let-7c 2.411595e-03      14 miRNA  TRUE
15     hsa-miR-34b 2.396425e-03      15 miRNA  TRUE
16     hsa-miR-10b 2.389018e-03      16 miRNA  TRUE
17     hsa-miR-18a 2.352102e-03      17 miRNA  TRUE
18     hsa-miR-328 2.344313e-03      18 miRNA  TRUE
19     hsa-miR-143 2.331756e-03      19 miRNA  TRUE
20     hsa-miR-214 2.313317e-03      20 miRNA  TRUE
21     hsa-miR-152 2.283654e-03      21 miRNA  TRUE
22    hsa-miR-135b 2.273221e-03      22 miRNA  TRUE
23     hsa-miR-195 2.270405e-03      23 miRNA  TRUE
24 hsa-miR-125a-5p 7.286119e-04      24 miRNA FALSE
25  hsa-miR-342-3p 5.469969e-04      25 miRNA FALSE
26      hsa-let-7a 4.714080e-04      26 miRNA FALSE
27  hsa-miR-769-5p 4.352923e-04      27 miRNA FALSE
28  hsa-miR-361-5p 3.802039e-04      28 miRNA FALSE
29  hsa-miR-142-3p 3.341059e-04      29 miRNA FALSE
30  hsa-miR-34c-5p 2.341773e-04      30 miRNA FALSE
31 hsa-miR-146b-5p 1.953439e-04      31 miRNA FALSE
32    hsa-miR-449a 1.556556e-04      32 miRNA FALSE
33 hsa-miR-199a-5p 1.228826e-04      33 miRNA FALSE
34     hsa-miR-489 1.197518e-04      34 miRNA FALSE
35       hsa-miR-9 9.189293e-05      35 miRNA FALSE
36    hsa-miR-30a* 9.030027e-05      36 miRNA FALSE
37    hsa-miR-135a 8.225978e-05      37 miRNA FALSE
38    hsa-miR-181c 7.444931e-05      38 miRNA FALSE
39    hsa-miR-378* 7.208481e-05      39 miRNA FALSE
40     hsa-miR-30c 7.006724e-05      40 miRNA FALSE
41      hsa-let-7f 6.601124e-05      41 miRNA FALSE
42  hsa-miR-483-3p 5.512170e-05      42 miRNA FALSE
43  hsa-miR-342-5p 5.253228e-05      43 miRNA FALSE
44  hsa-miR-501-3p 4.575108e-05      44 miRNA FALSE
45     hsa-miR-101 4.479886e-05      45 miRNA FALSE
46  hsa-miR-574-3p 4.081944e-05      46 miRNA FALSE
47     hsa-miR-26a 3.510984e-05      47 miRNA FALSE
48     hsa-miR-103 3.103517e-05      48 miRNA FALSE
49     hsa-miR-378 2.822501e-05      49 miRNA FALSE
50     hsa-miR-99b 2.788255e-05      50 miRNA FALSE
51  hsa-miR-590-5p 1.735067e-05      51 miRNA FALSE
52  hsa-miR-362-5p 1.092474e-05      52 miRNA FALSE
53      hsa-miR-9* 2.842895e-06      53 miRNA FALSE
54  hsa-miR-142-5p 1.520914e-06      54 miRNA FALSE
55    hsa-miR-101* 1.019211e-06      55 miRNA FALSE
```

```
56    hsa-miR-30e* 7.906392e-07       56 miRNA FALSE
57     hsa-miR-650 4.129138e-07       57 miRNA FALSE
```

Using RWRMTN in a workflow in other environments

In other environments, the similar procedure could be carried out by just changing the syntax of calling a CyREST API request and using the appropriate libraries to handle the retrieved outputs in JSON format. For example:
With the following commands for calling the CyREST API request in R:

```
###Step 1: Import library and prepare the parameters to pass to RWRMTN:
Select datasets (miRTargetDB, miR2DiseaseDB), the disease of interest (MIM114480: Breast c
ancer) and the candidate miRNAs list
library(httr)
library(jsonlite)

parameters <- list(
  diseaseOMIMID= "MIM114480",#OMIM ID of Breast cancer
  listOfmiRNAs= lor,
  miRTargetDB= "miRWalk",
  miR2DiseaseDB= "HMDD"
)
###Step 2: Send POST request to Cytosacpe CyREST API
request_body_json <- toJSON(parameters)
res <- POST("http://localhost:1234/RWRMTN/v1/rank", body = login, encode="json")
y<-httr::content(res,"text", encoding = 'UTF-8')
###Step 3: Parse JSON object of result to output
Output <- fromJSON((y))
```

Equivalent procedure in Python (use requests library) (python_callCyRestAPI.ipynb in Case Study folder)

```
###Step 1: Import library and prepare the parameters to pass to RWRMTN
Select datasets (miRTargetDB, miR2DiseaseDB), the disease of interest (MIM114480: Breast c
ancer) and the candidate miRNAs list (lor.txt)
import requests
x = [line.rstrip('\n') for line in open('lor.txt')]
lor=','.join(x)
parameters = {
  ‘diseaseOMIMID’= ‘MIM114480’ #OMIM ID of Breast cancer
  ‘listOfmiRNAs’= lor,
  ‘miRTargetDB’= ‘miRWalk’,
  ‘miR2DiseaseDB’= ‘HMDD’,
  'backProb':0.5,
  'subnetWeight':0.5
}
###Step 2: Send POST request to Cytosacpe CyREST API
res=requests.post(url="http://localhost:1234/RWRMTN/v1/rank", json=parameters)
###Step 3: Parse JSON object of result to output
Output = res.json()
print(Output)
```

Equivalent procedure in Bash (use curl - available in the Help/Automation/CyREST API) (curl_linux_callCyRestAPI.sh and lor.txt files in Case Study folder)

```
###Step 1: Read candidate miRNAs list in text file (file lor.txt) and make JSON object for
body of API request:
selecting datasets (miRTargetDB, miR2DiseaseDB), the disease of interest (MIM114480: Breas
t cancer) and the candidate miRNAs list.
vars=($(awk -F= '{print $1}' lor.txt))
var=$(IFS=',';echo "${vars[*]}";IFS=$' \t\n’)
echo '{"diseaseOMIMID": "MIM114480","miRTargetDB": "miRWalk", "miR2DiseaseDB":"HMDD",
"backProb":0.5, "subnetWeight":0.5}'| jq --arg v "$var" '. +
{"listOfmiRNAs":$v}’>para.json

### Use CURL of linux to make API request. The result of curl is stored in result.csv.
curl -X POST --header 'Content-type: application/json' --header 'Accept:
application/json' -d "@para.json" 'http://localhost:1234/RWRMTN/v1/rank' -o result.csv

###Step 2: Use jq tool to retrieve information of result.csv. For example, take the
rnaName:
```

```
Output = echo result.csv | jq ‘.[].rnaName’
```

IV. Case study: Prediction of lung cancer-associated miRNAs

Examining RWRMTN for different diseases rather than breast cancer also shows potential. In this section, we demonstrate one more use case of lung cancer, the leading cause of cancer-related deaths, using Cytoscape menu.

Step 1: Load datasets

We loaded the same datasets as those for breast cancer (i.e., TargetScan (Lewis, et al., 2003) and miR2Disease (Jiang, et al., 2009)). Then, in next step, we choose lung cancer to rank candidate miRNAs associated with that disease and have a quick look at the result.

Step 2: Rank candidate miRNAs

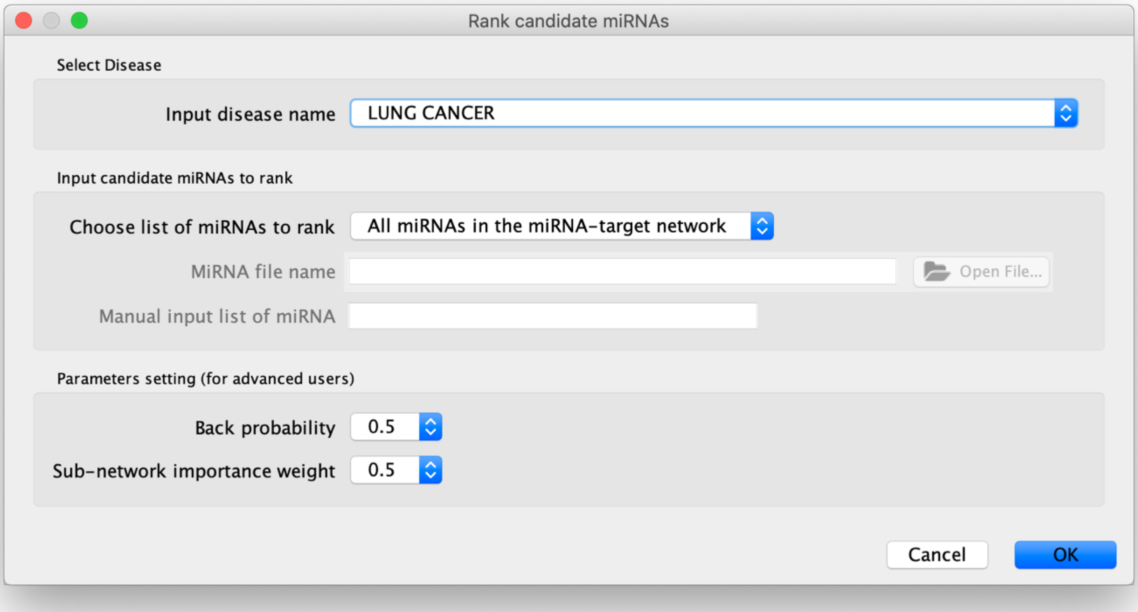

Step 3: Search Evidences

The top ten of result in Step 2 are further investigated by "RWRMTN Search Evidences" function:

shared name	name	Score	Rank	Type	Known	PubMed (PudMedIDs)
hsa-miR-137		0.01136250...	1	miRNA	☑	
hsa-miR-124		0.01129747...	2	miRNA	☑	
hsa-miR-29a		0.01065894...	3	miRNA	☑	
hsa-miR-29c		0.01065894...	4	miRNA	☑	
hsa-miR-29b		0.01065894...	5	miRNA	☑	
hsa-let-7g		0.01065010...	6	miRNA	☑	
hsa-let-7c		0.01065010...	7	miRNA	☑	
hsa-let-7f		0.01065010...	8	miRNA	☑	
hsa-let-7a		0.01065010...	9	miRNA	☑	
hsa-let-7d		0.01065010...	10	miRNA	☑	
hsa-let-7b		0.01065010...	11	miRNA	☑	
hsa-let-7e		0.01065010...	12	miRNA	☑	
hsa-miR-19a		0.01053038...	13	miRNA	☑	
hsa-miR-128		0.01047819...	14	miRNA	☑	
hsa-miR-17		0.01042976...	15	miRNA	☑	
hsa-miR-20a		0.01042976...	16	miRNA	☑	
hsa-miR-1		0.01027269...	17	miRNA	☑	
hsa-miR-34a		0.01016995...	18	miRNA	☑	
hsa-miR-34c-5p		0.01016995...	19	miRNA	☑	
hsa-miR-372		0.01012160...	20	miRNA	☑	
hsa-miR-221		0.00997750...	21	miRNA	☑	
hsa-miR-222		0.00997750...	22	miRNA	☑	
hsa-miR-34b		0.00997152...	23	miRNA	☑	
hsa-miR-183		0.00996808...	24	miRNA	☑	
hsa-miR-18a		0.00983121...	25	miRNA	☑	
hsa-miR-126		0.00972823...	26	miRNA	☑	
hsa-miR-506		0.00168247...	27	miRNA	☐	21726609, 24469051, 26341493, 27893417, 28405738, 30002440, 30535506, 30985742
hsa-miR-3163		0.00124109...	28	miRNA	☐	26482610
hsa-miR-4500		0.00103471...	29	miRNA	☐	
hsa-miR-4458		0.00103471...	30	miRNA	☐	28603287
hsa-let-7i		0.00103471...	31	miRNA	☐	21622546
hsa-miR-98		0.00103175...	32	miRNA	☐	21622546, 22862169
hsa-miR-520d-5p		9.30147779...	33	miRNA	☐	
hsa-miR-524-5p		9.30147779...	34	miRNA	☐	
hsa-miR-19b		9.15002558...	35	miRNA	☐	
hsa-miR-548c-3p		9.06775972...	36	miRNA	☐	

A total of 26 known miRNAs have already known to be associated with Lung cancer so after ranking, they have taken the position from 1st to 26th.

The top ten candidate miRNAs are ranked from 27th to 36th. We subsequently have a look at those miRNAs. Of them, five miRNAs were found with evidence of their association with lung cancer:

- "hsa-miR-506" has the highest rank among the candidate miRNAs and also the biggest number of evidences found. A total of eight studies were found in PubMed which mention miR-506 and lung cancer. In 2011, a study (PubMed ID: 21726609) explored the underlying miRNA involvement in lung carcinogenesis by experiments and found that the expression of miR-506 was reduced in human bronchial epithelial cells (16HBE-T) transformed malignant cells compared with 16HBE normal cells. These findings revealed that miR-506 acts as an anti-oncogenic miRNA in malignantly transformed cells. Four years later, a study (PubMed ID: 24469051) demonstrated that miR-506's role as mediates cross talk between three crucial elements of tumorigenesis: the tumor suppressor p53, nuclear factor-kB (NF-kB) and reactive oxygen species (ROS) based on experiments on 156 lung cancer patients. After that, from 2016 to 2019, different examinations involving miR-506 have been implemented in order to explore its role in oncogene (PubMed IDs: 26341493, 27893417, 28405738, 30002440, 30535506 and 30985742). For example, the study (PubMed ID: 26341493**)** observed miR-506 expression levels of different tissues and tumor types. They reported that the expression of miR-506 in the plasma samples was significantly lower in Lung cancer patients compared to healthy individuals. Interestingly, in another study (PubMed ID: 28405738**),** they found out that miR-506's expression in peripheral blood is reduced in lung, breast, NPC, pancreatic neoplasms but increased only in colorectal cancer. Besides, non-small cell lung cancer, the most popular type of lung cancer, has two studies on evaluation of miR-506's regulation in progression (PubMed ID: 27893417) and gefitinib sensitivity (PubMed ID 30535506). Recently, a study (PubMed ID: 30002440) has proved the combination of miR-506 and miR-143 inhibit lung cancer cell cycle progression and angiogenesis.
- "hsa-miR-3163" was involved in a study of Spk2 in non-small cell lung cancer (PubMed ID: 26482610). The result is shown that Meg3 and miR-3163 may coordinate suppression of translation of Skp2 mRNA in non-small cell lung cancer cells to inhibit the cell growth.
- "hsa-miR-4458" is concluded as tumor suppressor with direct target Lin28B by research of human lung cancer cells (PubMed ID: 28603287) First, this study also used database TargetScan to identify oncogene Lin28B. Then validating by RT-PCR in 40 human lung cancer tissues and matched peritumoral tissues, it was shown that the overexpression of mir-4458 signficantly decreased the protein levels of Lin28B in the cells, and inhibited the cell growth and colony formation.
- "hsa-let-7i" expression level is compared using Student t-test to differentiate squamous cell carcinoma from adenocarcinoma in 31 non-small cell lung cancer transthoracic needle aspiration specimens in the study (PubMed ID: 21622546**)**.
- "hsa-miR-98" was also investigated and reported as upregulating expression level in adenocarcinoma specimens in a study (PubMed ID: 21622546**)**. Besides, another research with PubMed ID 22862169 used miR-98 expression to prove that human lung cancer cell line SPC-A1 contains cells with characteristics of cancer stem cells.

Finally, a total of five in the top ten candidate miRNAs has been found the direct or indirect evidence of their association with lung cancer.

Step 4: Visualize

The top ten candidate miRNAs (ellipse nodes) with evidence by PubMed IDs (green parallelogram nodes) and 26 known lung cancer-associated miRNAs (triangle nodes) were selected for visualization.

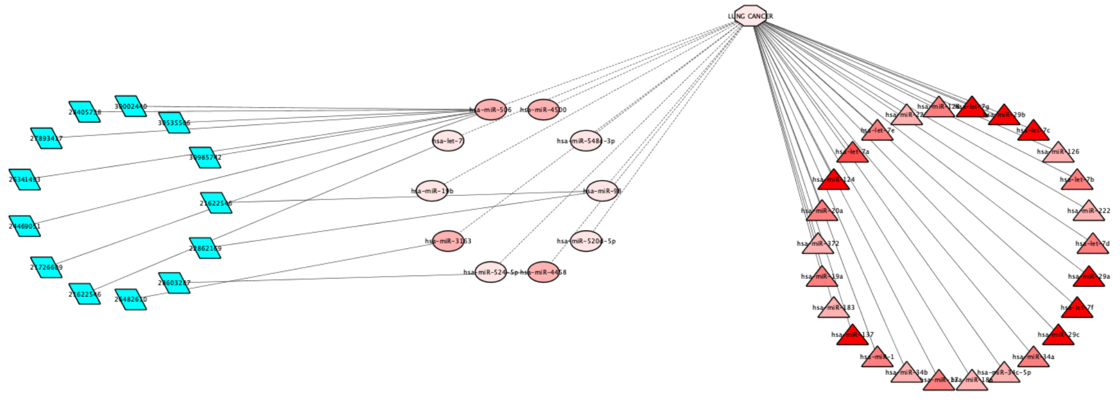

V. Reference

Dweep, H., *et al.* miRWalk - Database: Prediction of possible miRNA binding sites by "walking" the genes of three genomes. *Journal of Biomedical Informatics* 2011;44(5):839-847.

Enerly, E., *et al.* miRNA-mRNA Integrated Analysis Reveals Roles for miRNAs in Primary Breast Tumors. *PLOS ONE* 2011;6(2):e16915.

Jiang, Q., *et al.* miR2Disease: a manually curated database for microRNA deregulation in human disease. *Nucleic acids research* 2009;37(suppl 1):D98-D104.

Lewis, B.P., *et al.* Prediction of Mammalian MicroRNA Targets. *Cell* 2003;115(7):787-798.

Li, Y., *et al.* HMDD v2.0: a database for experimentally supported human microRNA and disease associations. *Nucleic Acids Research* 2014;42(D1):D1070-D1074.
